# Long-read genome sequencing and multi-omics in aging and neurodegeneration

**DOI:** 10.1101/2025.10.10.25337775

**Published:** 2025-10-29

**Authors:** Tanner D Jensen, Yann Le Guen, Lia Talozzi, Sherry Yang, John Gorzynski, Andrés Peña-Tauber, Ilaria Stewart, Alexis Ferrasse, Daniel Nachun, Maggie T Arriaga, Justin Lee, Rafael Catoia Pulgrossi, Junyoung Park, Jingyu Zhang, Anthony D. Wagner, Elizabeth C. Mormino, Kathleen L. Poston, Victor W. Henderson, Zihuai He, Tony Wyss-Coray, Stephen B Montgomery, Euan A Ashley, Michael D. Greicius

**Affiliations:** 1 Department of Genetics, Stanford University School of Medicine, Stanford, CA; 2 Quantitative Sciences Unit, Department of Medicine, Stanford University School of Medicine, Stanford, CA; 3 Department of Neurology and Neurological Sciences, Stanford University School of Medicine, Stanford, CA; 4 Department of Medicine and Surgery, University of Parma, Parma, Italy; 5 Department of Bioengineering, Stanford University, Stanford, CA; 6 Division of Cardiovascular Medicine, Department of Medicine, Stanford University School of Medicine, Stanford, CA; 7 Department of Pathology, Stanford University School of Medicine, Stanford, CA; 8 Department of Psychology, Stanford University, Stanford, CA, USA; 9 Department of Epidemiology and Population Health, Stanford University, Stanford, CA, USA; 10 The Phil and Penny Knight Initiative for Brain Resilience, Stanford University, Stanford, CA, USA

## Abstract

Structural variants (SVs) are a major source of genetic variation yet remain underexplored in healthy aging and neurodegenerative diseases. We performed nanopore long-read genome sequencing (lrGS) on 551 deeply-phenotyped individuals from Stanford’s Aging and Memory Study and Alzheimer’s Disease Research Center, generating a comprehensive SV map integrated with matched methylation, transcriptomic, and proteomic data. Over 60% of SVs identified by lrGS were not detected with short-read WGS, including many poorly tagged by single-nucleotide variants (SNVs). We discovered >60,000 SV-QTLs across molecular traits and showed that SVs were more likely than SNVs to be fine-mapped as causal. Colocalization with Alzheimer’s and Parkinson’s disease GWAS implicated SVs at multiple loci, including *TMEM106B*, *BIN3*, and *NBEAL1*. Multi-omic outlier enrichment and Bayesian modeling prioritized rare functional SVs near known risk genes. Combined, these data reveal widespread regulatory SVs in healthy aging and neurodegeneration, underscoring the importance of lrGS in deciphering complex genetic architecture.

## Introduction

Structural variants (SVs), defined as genomic alterations ≥50 base pairs including deletions, insertions, inversions, and complex rearrangements, represent a major source of genetic diversity and disease risk. SVs span larger genomic segments than single-nucleotide variants (SNVs) and are disproportionately enriched for functional effects on gene regulation, dosage, and chromatin structure (Collins et al. 2020; Eichler 2019). Yet much of their contribution to human disease remains uncharacterized, due to historical limitations in detection by short-read sequencing (Chaisson et al. 2019; Ebert et al. 2021).

Neurodegenerative disorders, such as Alzheimer’s disease (AD) and Parkinson’s disease (PD), exemplify these challenges. Recent genome-wide association studies (GWAS) have greatly expanded the list of loci associated with risk: the largest AD meta-analysis to date identified 75 loci implicating endocytic and immune pathways (Bellenguez et al. 2022), while a recent multi-ancestry meta-analysis of PD revealed 90 independent risk variants across diverse populations (Kim et al. 2024). A limited number of studies have examined SV associations with PD (Billingsley et al. 2023), AD (Vialle et al. 2025; Wang et al. 2025), and related dementias (Xu et al. 2024) using short-read whole-genome sequencing (srGS). In AD, one of the strongest examples of an SV tagged by the lead GWAS single-nucleotide variant (SNV) is a 316 bp Alu insertion in the 3’ UTR of *TMEM106B* (Chemparathy et al. 2024; Vialle et al. 2025; Bellenguez et al. 2022). Despite these initial insights, the degree to which SVs contribute to GWAS associations remains unknown.

Long-read genome sequencing (lrGS) is beginning to provide enhanced understanding of the contribution of SVs to human diseases. Recent projects, including the Human Genome Structural Variation Consortium (HGSVC) (Ebert et al. 2021), the Human Pangenome Reference Consortium (HPRC) (Nurk et al. 2022), the 1000 Genomes Project (Gustafson et al. 2024), and All of Us (Mahmoud et al. 2024), have shown that short-read pipelines miss more than half of SVs, especially in GC-rich, duplicated, or complex loci. These efforts have begun to reveal the spectrum of SV diversity and demonstrate the relevance of SVs detected with lrGS to both population genomics and disease biology (Logsdon, Vollger, and Eichler 2020).

Here, we present a population-scale long-read sequencing and multi-omics dataset from aged individuals with and without neurodegenerative disease. By integrating structural variant calls with methylation, single-cell transcriptomics, and proteomics, we uncover SVs that influence molecular traits and underlie unresolved GWAS signals in aging and neurodegeneration.

## Results

### Long-read sequencing of a deeply phenotyped neurodegenerative disease cohort shows SVs at GWAS loci

**Figure 1.**
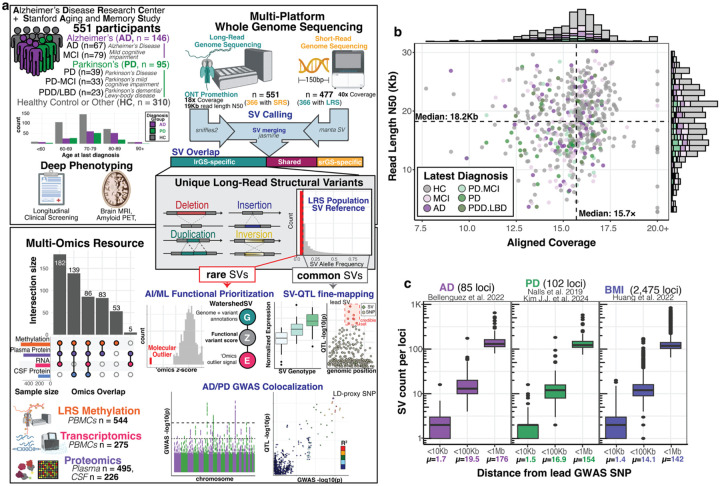
Long-read sequencing and multi-omics in healthy aging and neurodegenerative disease. **a**, Illustrative outline of paper analyses. Data from aging individuals from the Stanford Alzheimer’s Disease Research Center (ADRC) and the Stanford Aging and Memory Study (SAMS) were analyzed using long-read sequencing. ADRC and SAMS cohorts contain a wealth of deep phenotype data, including initial neuropsychological assessment and, for ADRC, yearly clinical visits, along with MRI and PET scans, proteomics from plasma and CSF, and transcriptomics from PBMCs. Long-read sequencing revealed structural variants (SVs) (many not detected with short-read sequencing) that we interpreted using matched multi-omics data. Functional rare SVs were prioritized with WatershedSV, and functional common SVs were identified through QTL fine-mapping. Colocalizing QTL associations with Alzheimer’s and Parkinson’s disease GWAS reveals functional SVs that are likely causal at neurodegeneration risk loci. **b**, Sequencing summary metrics of 551 long-read genomes sequenced on R9.4 flowcells on the PromethION device. Colored by diagnosis group, HC=healthy control, AD=Alzheimer’s disease, MCI=mild cognitive impairment, LBD=Lewy body dementia, PD=Parkinson’s disease. **c**, Burden of SVs from Sniffles2 joint genotyping near GWAS lead SNVs at various distance thresholds for 3 different GWAS phenotypes. For PD, SNV-union between Nalls et al. and Kim JJ et al was used. BMI=body mass index.

We performed nanopore-based lrGS on 551 individuals recruited as part of the Stanford Aging and Memory Study (SAMS only; n=125), the Stanford Alzheimer’s Disease Research Center (ADRC) (n=391), or co-enrolled in both (n=35). Participants were characterized as cognitively normal seniors or grouped based on clinical neurodegenerative disease diagnoses (mild cognitive impairment MCI, Alzheimer’s disease, Parkinson’s-MCI, Parkinson’s disease, Lewy body dementia) supported by biomarker data. Participants spanned an age range of 36–88 years old at recruitment (mean = 70.7; std = 8.0), with 261 males and 290 females (Figure 1a, Extended Data Table 1). Participants underwent extensive cognitive assessments, with multiple biomarker data types collected from plasma, cerebrospinal fluid (CSF), brain MRI, and amyloid PET. A subset of participants also had short-read whole genome (srGS) using MGI-sequencing (n=366), as well as SomaLogic proteomics from plasma (n=495) and/or CSF (n=226), and single-cell RNA sequencing from peripheral blood mononuclear cells (PBMCs) (n=275). For our study, DNA extracted from PBMCs was sequenced on the Oxford Nanopore PromethION to generate lrGS data, achieving a median coverage of 16× and read-length N50 of 18 kb after sample quality control (Figure 1b). Structural variants (SVs) and methylation were subsequently called from the long-read genome data (Methods). Population stratification analysis of common SVs (MAF > 2%) showed expected clustering by self-reported race and ethnicity (Extended Data Figure 1). Many SVs overlapped 85 known Alzheimer’s (Bellenguez et al. 2022) and 102 known Parkinson’s GWAS loci (Nalls et al. 2019; Kim et al. 2024). While a higher burden of SVs was observed within 100Kb of AD loci (mean = 19.5) relative to a large-scale control GWAS for BMI (Huang et al. 2022; mean = 14.1; p = 0.025), the overall pattern of SV burden near GWAS loci was broadly similar (Figure 1c).

### Most SVs discovered by long reads remain undetected by conventional short-read pipelines

**Figure 2.**
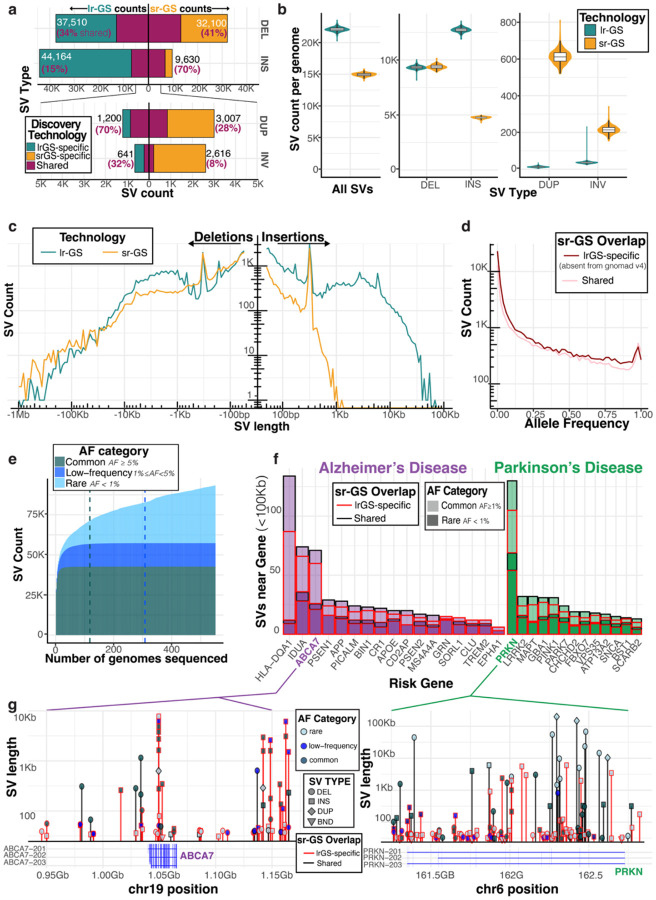
Long-read-specific structural variants across the frequency spectrum are found near neurodegeneration risk genes. **a,** In 366 matched individuals with both LRS and SRS genomes, count of structural variants identified by each technology after merging SVs with Jasmine. Percentages of shared SVs found in each technology that were also detected by the other technology are highlighted. **b,** Burden of SVs per genome comparing LRS Sniffles2 call set and SRS manta-paragraph call set. **c,** In the 366 matched individuals, count of deletions and insertions identified by each technology across variant length spectrum. **d,** Sniffles2 joint genotyping was used to ascertain allele frequencies for SVs. Counts of SVs shown across frequency spectrum, stratified by whether variant was specific to LRS (absent from short-read call set and additionally not found in SRS-based population resources gnomad v4), or also detected by SRS. **e,** Number of total SVs identified as a function of how many genomes were sequenced. Vertical lines show saturation point for common variants (AF ≥ 0.05) and for low-frequency variants (0.01 ≤ AF < 0.05). **f,** Count of SVs within 100Kb of a selection of well-known Alzheimer’s and Parkinson’s disease genes, stratified both by whether variant is rare or common and whether it was detected by short-read sequencing or not. **g,** Structural variant landscape in 100kb window around AD gene *ABCA7*, and PD gene *PRKN*. X-axis displays left break-point of SV and Y-axis displays variant length. Lollipops are shaped by SV type and colored by allele frequency, long-read-unique variants absent from SRS population references colored in red.

Among the 366 individuals with both srGS and lrGS data, we observed that the majority of lrGS insertions (85%) were unique to long-read, while 70% of srGS insertions were detected in long-read (Figure 2a). For deletions, the difference was more modest; 66% of lrGS deletions were unique to long-read, while 59% of srGS deletions were unique (Figure 2a). While most SVs are insertions and deletions, we also detected duplications and inversions. For these variants, srGS identified higher counts, particularly for duplications under 1kb (Extended Data Figure 1). The increased calling of inversions and duplications in srGS may be related to the higher coverage srGS to identify read-depth changes and break points, false positives which are more prevalent in srGS, or the long-read call set splitting some duplications as insertions.

On average, lrGS yielded ~22,400 SVs per genome, compared to ~15,000 from srGS (Figure 2b). Individuals with non-European ancestry showed a higher median number of SVs per genome compared to European participants (Extended Data Figure 1). The length distribution of long-read SVs exhibited pronounced peaks at ~300 bp, ~2.5 kb, and ~6 kb, corresponding to Alu, SVA, and LINE-1 mobile elements (Audano et al 2019), while short-read detection dropped sharply for insertions >1 kb (Figure 2c).

Upon intersecting our lrGS call set with short-read-derived SVs in gnomAD v4 (Collins et al 2020), we found that over 50% of lrGS SVs were absent from population references; those SVs shared with reference datasets exhibited a similar frequency distribution but were primarily composed of deletions and short insertions (<1 kb). Among low-frequency and common SVs with minor allele frequency (MAF) >2%, we observed that short insertions and deletions (<150 bp) frequently exhibit low linkage disequilibrium (LD) (r^2^ < 0.3) with nearby SNVs, indicating poor tagging. Mid-sized SVs (150–1000 bp), particularly insertions, exhibited stronger LD with adjacent SNVs (Extended Data Figure 2). Furthermore, SVs with higher overlap fractions (OFP) with gnomAD v4 short-read SVs demonstrated improved SNV tagging (SNVs with highest LD with SVs), especially among mid-sized insertions (Extended Data Figure 3).

### Population-scale long-read sequencing saturates the discovery of common SVs in European populations

Population sequencing of 551 nanopore genomes enabled estimation of structural variant allele frequency. We used *Sniffles2* to jointly genotype SVs across the cohort and calculate allele frequencies (AFs). Additionally, we queried existing srGS-based population references (i.e. gnomAD v4) to examine the extent of overlap with our lrGS calls (Methods). We define lrGS-specific as novel variants absent from these srGS resources and not detected in our cohort’s matched short-read call set. We found 54% of common SVs (minor allele frequency ≥ 5%) were specific to lrGS; this increased to 64% for rare and low-frequency variants (MAF < 5%). Overall, 40% of SVs in our cohort were rare (MAF < 1%) (Figure 2d), demonstrating the necessity of population-scale lrGS sequencing to establish the AF of novel lrGS variants. We share allele frequencies and coordinates of the SVs we discovered as a resource to help identify and filter out SVs that are tolerated in healthy aging (Table S1, see Data Availability).

To determine if the size of our cohort was sufficiently large to detect most common SV alleles, w e performed a down-sampling analysis. By calculating the number of SVs detected as a function of genomes sampled, we found that counts of common variants (AF ≥ 5%) saturated at 67 genomes. For low-frequency variants (1% ≤ AF < 5%), this saturation occurred after 272 genomes. As expected, counts of rare variants continued to increase with each genome sequenced at a rate of about 45 variants per genome (Figure 2e). Extrapolating from these saturation points, we expect we would need to sequence 12,000 genomes to achieve saturation of variants above an allele frequency of 0.1% (Extended Data Figure 1). Together, these data indicate that with 551 genomes, we have achieved comprehensive coverage of common and low-frequency SV alleles in our mostly-European population sample.

Having discovered lrGS-specific variants across the frequency spectrum, we investigated whether these variants could be impacting risk genes for neurodegenerative disease. We intersected our SV calls with a selection of 30 well-known Alzheimer’s and Parkinson’s disease genes, and found these genes had a median of 20 SVs (range 6–134) in a 100Kb window; overall 46.4% were rare and 71% were novel to lrGS (Figure 2f). The structural variant landscape of two genes of interest, *ABCA7* for AD, which is known to harbor complex structural rearrangements, and *PRKN* for PD, a long gene with known deletion and duplication alleles, depicts the density of lrGS-discovered variants across both length and frequency spectrum, highlighting the discovery power of performing long-read sequencing to find novel variants near disease genes (Figure 2g).

### Integration of multi-omics and long-read sequencing reveals outliers enriched near rare SVs

**Figure 3.**
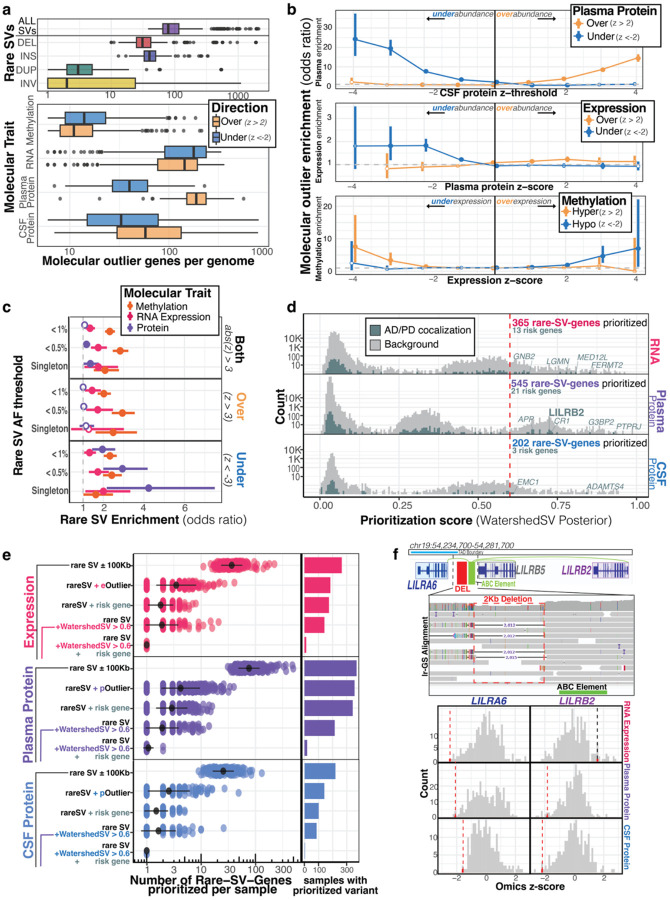
Molecular outliers prioritization of functional rare structural variants impacting neurodegenerative disease genes. **a,** Burden of molecular outliers per individual across molecular traits. Rare SV outlier genes are defined by having a rare SV within 100Kb of a gene. Methylation outliers are defined by having a methylation outlier within 10Kb of a gene. **b,** Pairwise multi-omic outlier enrichments showing the concordance of outliers across tissues and layers of regulation. X-axis depicts the z-score threshold to define outliers--negative values for under-outliers (z-score < -x), positive values for over-outliers (z-score > x). Y-axis displays Fisher’s exact test odds ratio of observing an outlier in a comparison ome (y-axis label) given an outlier was observed in a discovery ome (x-axis label). Solid points represent nominally significant enrichments, white-filled points are not significant. **c,** Enrichment from Fisher’s exact test of rare SVs within 100Kb of molecular outlier genes at various AF thresholds. Singleton SVs are variants only identified in a single genome. Y-axis stratified by outlier direction. “Both” represents outliers in either tail, while “under” and “over” represent outliers in negative and positive direction, respectively. White-filled points represent enrichments that were not nominally significant. **d,** Distribution of WatershedSV posteriors across RNA expression, plasma protein, and CSF protein. Log-scaled y-axis shows the histogram of rare-SV-genes scored across posterior threshold. Line at posterior = 0.6 represents the prioritization cut-off used to label functional rare variants. Risk genes based on colocalizations of AD and PD GWAS are highlighted. **e,** Prioritization scheme for identifying disease-relevant functional rare variants. Number of SV-gene pairs per genome are triaged down based on additional filters including, if a gene is an outlier abs(z-score)>2 (rare SV + outlier), whether a gene is in a risk gene list based on AD/PD GWAS colocalizations (rare SV + risk gene), whether a gene was highly prioritized with Watershed (rare SV + WatershedSV > 0.6), and a combination of both Watershed prioritization and colocalization evidence (rare SV + WatershedSV > 0.6 + risk gene). Bar plot shows the number of individuals with at least 1 rare-SV-Gene pair prioritized with a given filter. **f,** Example of a rare, non-coding deletion prioritized by WatershedSV. A rare 2 kb deletion in the LILRB gene cluster on chr19 was called in a single SAMS individual as seen in the IGV screenshot showing read alignment pile-up. The deletion was proximal to an ABC element linked to nearby *LILRA6* and *LILRB2* genes. *LILRB2* is a known Alzheimer’s risk gene based on GTEx eQTL colocalizations. Across multiple ‘omes, the deletion-carrier sample was an under-outlier in both LILRA6 and LILRB2 as seen in omics z-score histograms. Vertical line represents the deletion-carrier’s z-score, colored in red if the carrier is in the bottom 1% tail of the distribution.

To interpret the functional consequences of the rare variants we discovered with lrGS, we leveraged existing multi-omics data available for ADRC and SAMS participants. We used methylation, RNA, and protein-level molecular outliers to inform the influence of a rare variant on molecular traits (Ferraro et al. 2020; Li et al. 2023; Chui et al. 2025). We discovered 9,211 expression and 38,074 protein outliers across 4,659 genes and 6,004 proteins, respectively. Using lrGS, we called CpG methylation and detected a total of 14,462 methylation outliers across 6,641 unique regions (Extended Data Figures 4a). Summarizing this outlier information, we found that a typical genome has a median burden of 79 genes with a nearby (<10Kb) rare SV, 25 genes with methylation outliers, 354 genes with RNA expression outliers, and 229 and 92 proteins with abundance outliers in plasma and CSF, respectively (Figure 3a).

Many of these molecular outliers were shared across various tissues and layers of regulation. We found that over-expression outlier genes were strongly enriched for having hypomethylation outliers (odds-ratio (OR)=5.21, p-value=9.9E-8); conversely, under-expression outliers were enriched for hypermethylation outliers (OR=2.82, p-value=1.1E-3), supporting the canonical model that promoter methylation is associated with silencing of genes. Comparing expression to protein levels, we observed enrichment of under-expression outliers with under-abundance outliers of plasma protein (OR=1.6, p-value=1.3E-8), and weaker enrichment for over-expression with protein over-abundance (OR=1.15, p-value=6.6E-3), indicating that under-expression was a stronger predictor of protein abundance than overexpression. Finally, we demonstrate replicability across two different tissues, CSF and plasma; CSF outliers were strongly enriched near plasma outliers in concordant directions (under-under: OR=20.3, p-value<1E-32; over-over: OR=8.7, p-value<1E-32) (Figure 3b).

We assessed if molecular outliers were enriched near rare SVs as reported in prior SV studies (Chiang et al. 2017; Jensen et al. 2024). Here, we replicated the expression outlier enrichment of rare SVs in our ADRC cohort (OR=1.34, p-value=7.9E-4) and further show rare SVs are also enriched near methylation and protein outliers (methylation OR=2.32, p-value<1E-32; plasma protein OR=1.35, p-value=0.034). When stratifying enrichments by outlier direction, we observe that expression and methylation were enriched in both directions, whereas plasma protein was only enriched for under-abundance (OR = 1.96, p-value = 1.92E-5), as was previously observed for the enrichment of rare SNVs (Li et al. 2023). We saw the strongest enrichments at lower allele frequency thresholds for rare SVs, particularly singleton variants, where rare SVs were 4x more likely to be seen near protein under-expression outliers compared to inliers (Figure 3c). When calculating type-specific enrichments for rare SVs, we observed that insertions were most enriched for driving hypermethylation outliers, and deletions were particularly enriched for under-abundance protein outliers (Extended Data Figure 4b). In general, we observed higher enrichments at higher absolute z-score thresholds (Extended Data Figure 4c) and for SVs overlapping the gene body. However, we detected long-range enrichment of rare SVs on methylation and expression as far out as 500Kb from the gene (Extended Data Figure 5d).

### Outliers prioritize functional SVs near neurodegeneration risk genes

We applied WatershedSV, a hierarchical Bayesian model that integrates outlier omics signals with genomic annotations, to prioritize potentially functional rare SVs. Variants were scored separately using three WatershedSV models for RNA expression, plasma protein levels, and CSF protein levels as each model prioritizes different annotations (Supplementary Figures 1–3) We identified 365, 545, and 202 prioritized rare SV-gene pairs from RNA expression, plasma protein levels, and CSF protein levels, respectively (Watershed posterior probability > 0.6) (Table S2, Figure 3d).

We next applied additional filters to the WatershedSV-prioritized variants to prioritize those most likely contributing to AD or PD risk. SVs were categorized based on outlier status of proximal genes, WatershedSV prioritization score, and whether gene had eQTL or pQTL colocalization evidence from AD or PD GWAS (coloc PP4 > 0.5). Among colocalized risk genes, we found 13, 21, and 3 prioritized rare SV-gene pairs for expression, plasma protein, and CSF protein, respectively (Figure 3e). Notably, one such prioritized rare SV near *LILRB2*—an Alzheimer’s risk gene identified through GTEx eQTL colocalizations—was detected in plasma protein data. This rare SV deletes a 2kb non-coding region of the *LILRB* gene cluster, adjacent to a CTCF binding site and an ABC regulatory element linked to both *LILRA6* and *LILRB2*. The deletion was identified in a single individual from the SAMS cohort, and the deletion-carrier sample was a strong under-outlier in both *LILRA6* and *LILRB2* expression across multiple omics layers (Figure 3f).

### SV-QTL mapping across multi-omics discovers novel associations

**Figure 4.**
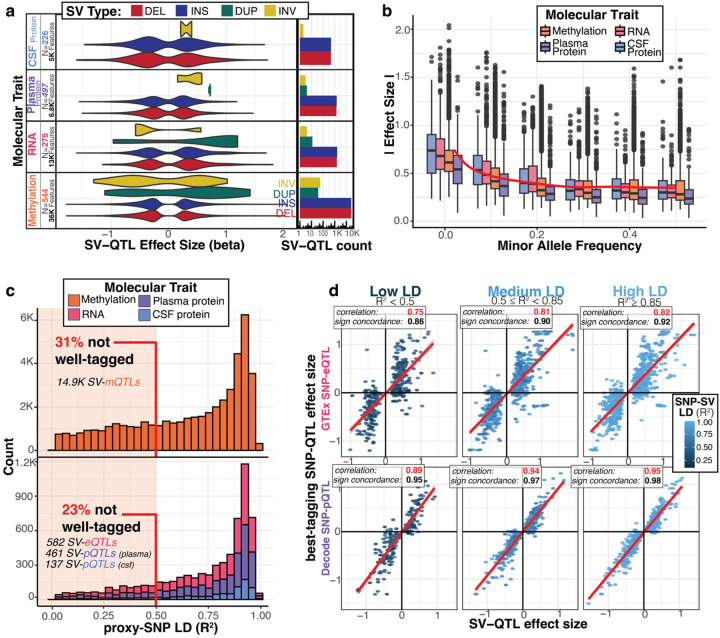
SV-QTL mapping identifies widespread regulatory structural variants not well-tagged by SNVs **a,** Count and effect size distribution of significant SV-QTL at an FDR threshold of 0.05. Number of samples and features per ome meeting expression and variance thresholds to be tested are also annotated. **b,** Absolute effect size of SV-QTLs stratified by molecular trait and minor allele frequency bins. Red trend line shows loess fit of the relationship between MAF and effect size across all traits. **c,** Histogram of R^2^ correlation between SV-QTL and its best-tagging SNV genotypes; SVs that are poorly tagged (R^2^ < 0.5) are highlighted. Methylation SV-QTLs plotted on separate axes due to the increased number of effects. **d,** Scatter plot comparing SV-QTL effect size to the best-tagging SNV-QTL effect size from two public QTL resources: GTEx eQTLs from whole blood compared to ADRC eQTLs and deCODE pQTLs from plasma to compare to ADRC plasma pQTLs. Scatter plots are split by the strength of LD between SV-QTL and best-tagging SNV. Correlation of SNV and SV betas and sign concordance of betas calculated for each LD bucket.

To prioritize low-frequency and common structural variants (MAF > 2%) that may be functional, we used SV genotypes from lrGS and the matched omics data to map molecular QTLs. At an FDR threshold of 0.05, we discovered 60,177 SV-QTLs for 16,201 unique features (molQTL targets) over our 4 molecular traits (Figure 4a, Extended Data Figure 6a, Table S3) (methylation: 53,112 SV-QTLs in 13,397 regions; expression: 3,202 SV-QTLs in 1,216 genes; plasma: 2,904 SV-QTLs in 1,149 aptamers; CSF: 959 SV-QTLs in 439 aptamers). While most QTLs we found were for insertions and deletions, when a QTL was detected for an inversion or duplication, it exhibited larger effect sizes. A typical QTL-associated SV affected 1.51 genes for expression and protein, and 2.36 regions for methylation. Similarly, a typical molQTL target had 2.5 associated SVs across expression or protein, and an average of 4 SV-QTLs for methylation (Extended Data Figure 6b). By overlapping QTL-associated SVs across molecular traits, we discovered SVs with effects across multiple molecular traits, including over 100 SVs which had a significant QTL in all 4 traits. (Extended Data Figure 6c)

62.1% of our SV-QTLs included a lrGS-specific SV, particularly for insertions where a majority (76%) of QTLs were lrGS-specific (Extended Data Figure 6d). Given the sample size of our lrGS dataset, we were able to ascertain low-frequency variants and include them in QTL mapping. When stratifying SV-QTL effect size by MAF, we observed that low-frequency SVs (MAF 2–5%) had significantly higher effect sizes compared to more common SVs (beta=0.26, p-value<1E-32) (Figure 4b). In addition to allele frequency, we also observed that longer variants had larger absolute effect sizes (Length>10Kb, beta=0.11, p-value<1E-32) (Extended Data Figure 6e). Since SVs can have long-range effects, we tested all SVs within a 1Mb window of the TSS/methylation region and observed that 16% of SV-QTLs were within 10Kb and 58% were within 100Kb. QTLs for methylation had closer-range effects compared to the other omes (p-value=9.4E-18), and across all molecular traits, closer SVs had significantly higher effects (log-distance slope=−0.022, p-value<1E-32), particularly those for SVs directly overlapping their molecular target (Extended Data Figure 6f,g).

To investigate the extent to which SV-QTLs represent novel associations, we investigated the LD of these SVs with nearby SNVs. For each SV-QTL, we defined an LD-proxy as the SNV with the highest genotype correlation with the SV. While most SVs were in high LD with their proxy SNV, we found that 32% of methylation SV-QTLs and 23% of expression and protein QTLs had *R*^2^ less than 0.5, representing over 16K unique SV-QTLs. Further, when comparing SV-QTL effect sizes from our QTL study to SNV-QTLs from GTEx v8 whole blood for eQTLs and deCODE genomics for plasma pQTLs, we observed higher correlation and sign concordance between SV-QTL effect sizes with proxy-SNV QTL effect sizes as a function of increasing LD (Figure 4d). This indicates that many previously reported QTL associations that relied only on SNV genotypes may be driven by an SV in high-LD.

### Fine-mapped structural variants underlie many QTL associations and exert larger effects than SNVs

**Figure 5.**
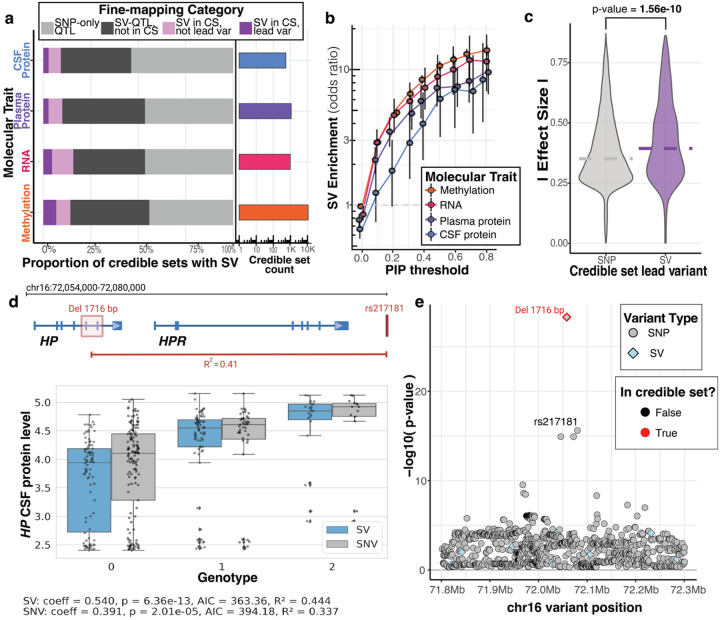
Fine-mapping reveals causal structural variants at QTL loci **a,** Count of credible sets for each molecular trait, as well as proportion of those credible sets that were fine-mapped to contain an SV. “SNV-only QTL” traits contained no significant SV-QTLs., “SV-QTL not in CS” traits had a significant SV-QTL, but the SV was not included in the credible set. “SV in CS, not lead var” traits contained an SV in one of their credible sets, but the variant with the highest PIP (lead variant) was an SNV. Finally, “SV in CS, lead var” traits were those where an SV was included in the credible set and an SV had the highest PIP. **b,** Enrichment of an SV being a causal variant compared to an SNV across multiple molecular traits. Causality is defined by having high PIP from SuSiE’s fine-mapping at various PIP thresholds. Odds ratio and confidence intervals from Fisher’s exact test are plotted. **c,** Absolute effect size distributions of SV-QTL credible sets stratified by whether an SV or an SNV was the highest pip lead variant. **d,** Schematic of the HP locus with 1.7 kb deletion marked as well as its best-tagging SNV rs217181. Box plot demonstrates SV genotype has a stronger association than SNV with *HP* protein levels in CSF**. e,** Fine-mapping of the *HP* locus confidently puts the deletion variant in the credible set by itself

To further identify which QTLs are likely driven by SVs instead of SNVs, we combined SV and SNV genotypes and performed statistical fine-mapping of QTLs with Sum of Single Effects (SuSiE). We labelled each association as to whether an SV was contained in the credible set and, additionally, if it had the highest Posterior Inclusion Probability (PIP) score. Prior work in GTEx using srGS estimated that SVs are likely causal at 3.5% to 6.8% of eQTLs, with SVs as the lead variant in 3.5% of eQTLs (Chiang et al. 2017). With our lrGS SV calls, we observed 12% of QTL associations having an SVs in the credible set, with 6% having an SV as the lead variant (Figure 5a, Table S4). 54% of these SVs contained in credible sets were novel to lrGS (Extended Data Figure 7a).

We observed that SVs were significantly enriched in fine-mapped credible variant sets as a function of PIP threshold (Figure 5b). At PIP thresholds >0.8, SVs were enriched in credible sets across each molecular QTL at rates up to 12x higher than SNVs. Across SV types, deletions and duplications were most enriched to be fine-mapped in credible sets (Extended Data Figure 7b), and longer SVs that disrupt more sequence were also more likely to be included (Extended Data Figure 7c). Further, if the fine-mapped credible set had an SV as the lead variant, we observed a significant increase in the effect size of the QTL (p-value=1.56E-10; Figure 5c). Credible sets with a lead SV tended to be smaller than those containing a lead SNV (Extended Data Figure 7d). Comparing fine-mapped SVs to other SV-QTLs not included in credible sets, we found lead SVs tended to be in lower LD with nearby SNVs (p-value<1E-32, Extended Data Figure 7e). Fine-mapped SVs in QTL credible sets were also more likely to disrupt functional regions compared to background SVs, as evidenced by a higher non-coding constraint score from Gnocchi for these variants (Chen et al. 2024) (Extended Data Figure 7f).

One AD-relevant fine-mapped variant is a large deletion in HP (chr16:72057133–72058849; 1,716 bp deletion), which spans two exons and results in a different protein isoform: a dimeric form (HP1) when both exons are deleted, showing reduced binding affinity to APOE and hemoglobin (Boettger et al. 2016), in contrast to the tetrameric form when no deletion is present, or the trimeric form when only one allele is affected. In our findings, the 1,716 bp deletion emerged as the lead variant regulating HP protein levels, surpassing the signals from nearby SNVs (Figure 5d,e).

### Colocalization with neurodegeneration GWAS nominates structural variants that may confer disease risk

**Figure 6.**
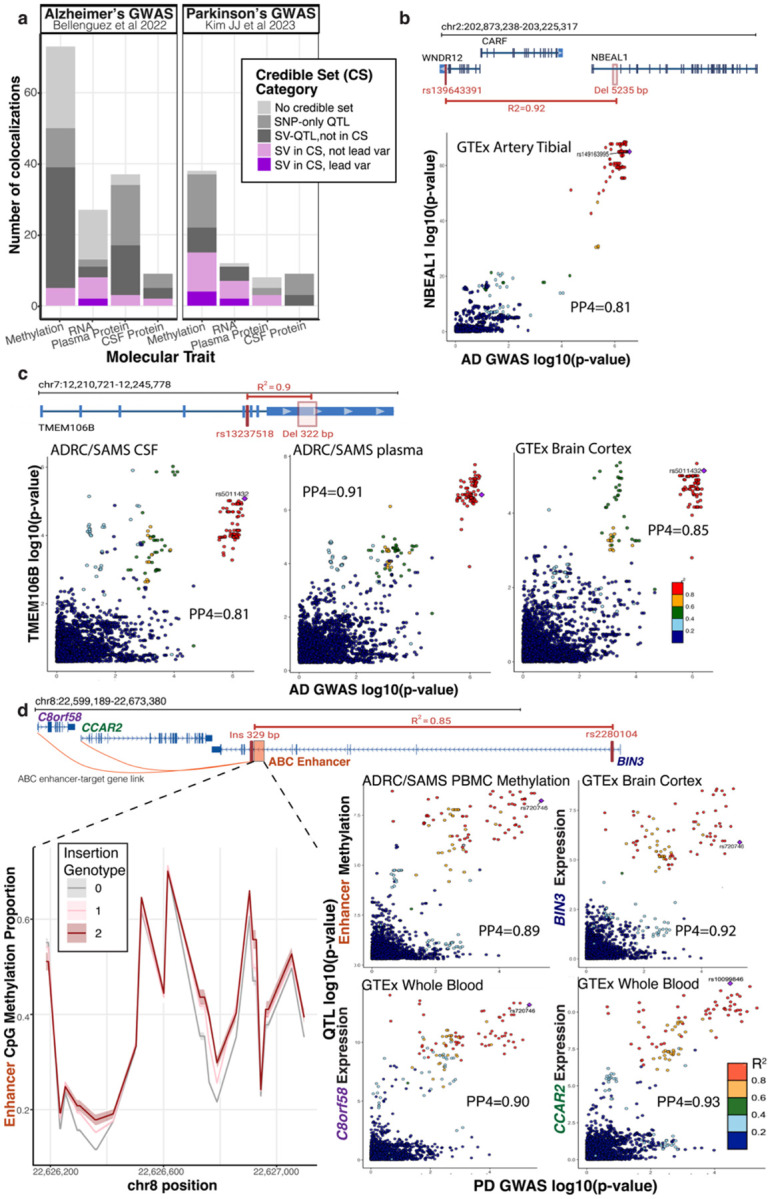
Colocalization across Alzheimer’s and Parkinson’s GWAS in loci with SVs tagging GWAS SNVs. Colocalization plots were generated showing PP4 values and highlighting the top GWAS SNVs at the intersection of the evaluated SNVs with ADRC molecular traits and GTEx expression data. **a**, Number of molecular traits colocalizing with neurodegenerative Alzheimer’s and Parkinson’s GWAS (posterior probability H4 > 0.5). **b**, Intronic deletion in *NBEAL1* in high LD with AD-associated SNV, shown alongside GTEx colocalization with *NBEAL1* expression in the tibial artery and AD GWAS. **c**, Alu element deletion in the 3′ UTR of *TMEM106B* in high LD with an AD-GWAS SNV, colocalizing with ADRC plasma and CSF *TMEM106B* protein levels and for expression in brain cortex from GTEx. **d**, Intronic Alu element insertion in *BIN3* in high LD with a PD-associated SNV, with locus-level colocalization involving ADRC methylation and GTEx brain expression brain cortex. Line plot shows mean and standard error estimate of methylation of CpGs within the enhancer across the 3 different genotype groups, showing individuals with higher copies of the *Alu* insertion display higher methylation and silencing of the regulatory element. ABC links enhancer to *BIN3*, and nearby genes *C8orf58* and *CCAR2,* whose expression both colocalized with PD risk from Kim JJ et al.

To assess the relevance of these molecular trait associations to neurodegenerative disease, we performed colocalization analyses with Alzheimer’s and Parkinson’s disease GWAS for all our ADRC and SAMS QTLs. We identified 146 and 67 colocalizations (PP H4 > 0.5) from 46 and 14 unique loci for AD (Bellenguez et al. 2022) and PD (Kim et al. 2024), respectively, across our four molecular traits (Figure 6a, Table S5, Table S6). Further, 20.2% of these colocalizations contained a fine-mapped SV in the QTL credible set, with 8 examples of the SV being the lead variant. All together, these represent 8 unique risk loci for AD and 4 for PD that are colocalized with a fine-mapped SV association, representing candidate GWAS signals that are driven by an SV (Extended Data Table 2).

We additionally colocalized GTEx-QTLs with high-tagging SVs to capture associations in tissues not profiled in this study (Supplementary Figure 4–5). An interesting locus emerged from these AD–GTEx colocalizations for the gene NBEAL1, a protein missing from both aptamer-based and Olink proteomics platforms. We found a large intronic deletion in NBEAL1 (chr2:203034349–203039584; 5,235 bp) that is in high LD with SNVs previously reported in AD (Bellenguez et al. 2022, rs139643391 on WDR12, R^2^ =0.92); small vessel disease (Chung et al. 2019; rs72932727 on ICAIL, R^2^=0.89); and ischemic stroke and white matter hyperintensities (Traylor et al. 2021; rs7934535 on NBEAL1, R^2^=0.82) (Figure 6b). QTL variants for NBEAL1 expression in the GTEx Tibial Artery tissue colocalized with Alzheimer’s GWAS (PP4=0.81) and across all neurovascular GWASs (small vessel disease PP4=0.74, stroke and white matter hyperintensities PP4=0.84) (Extended Data Figure 8).

We found additional candidate risk SVs from our ADRC molecular trait colocalizations that contained SVs in QTL credible sets. Of particular relevance is an Alu element insertion in TMEM106B (chr7:12242079–12242401; 322 bp insertion), well tagged by AD GWAS loci (rs13237518 R^2^=0.91) and previously reported (Chemparathy et al. 2024). Also present in the credible sets is an Alu element insertion in ACE (chr17:63488532; 291 bp insertion) (Supplementary Figure 6), which has been shown to affect isoform splicing (Wu et al. 2013) and may be a relevant locus for AD (R^2^=0.4 with rs4277405 reported in AD-GWAS, R^2^=0.62 with rs4309 in CSF proteomics GWAS) (Cruchaga et al. 2023). The SAMS/ADRC CSF protein levels of the corresponding SNVs in both genes showed strong colocalization with AD GWAS signals (PP4=0.96 ACE CSF protein levels, Supplementary Figure 7), TMEM106B protein levels (PP4= 0.81 CSF, PP4= 0.91 plasma), as well as GTEx expression (PP4=0.85 brain cortex, and Figure 6c).

An ADRC methylation signal colocalized with the *BIN3* Parkinson’s disease locus, where an intronic Alu insertion (chr8:22625890; 329 bp) acts as a methylation QTL for a nearby variable methylation segment (PP4 = 0.89) and is included in the fine-mapped credible set. This methylation segment overlaps a strong ABC enhancer, and individuals with more copies of the insertion exhibited higher rates of methylation across the enhancer CpGs (Fulco et al. 2019). Varying methylation at this regulatory element may modulate how this enhancer regulates its predicted target genes, including CCAR2 and C8orf58. We further observed the expression of these genes in GTEx whole blood, as well as BIN3 expression in cortex and cerebellum, also colocalized strongly with PD GWAS (Figure 6d).

High colocalization signals also emerged at the large ~1 Mbp inversion region spanning *MAPT* and neighboring genes. This region is primarily known for its role in PSP (rs62057121) (Wang et al. 2024), but also contains GWAS hits for both AD and PD. Within the inverted region, numerous SNVs are in high LD (Bowles et al. 2022); however, we also identified SVs encompassing *LINC02210-CRHR1*, *MAPT*, and *KANSL1*, as well as intergenic variants such as an SVA_67 (17:46237502–46238226, 724 bp deletion) located downstream of *LRRC37A*. Interestingly, across ADRC molecular traits, methylation regions and the expression of LRRC37 family genes show high colocalizations with disease risk. SVs are also present within the fine-mapped credible set—for example, LRRC37A2 (ENSG00000238083) shows strong colocalization with PP4 = 0.79 for AD and PP4 = 0.98 for PD (Supplementary Figures 8), with a lead SV (37 bp insertion) in the LRRC37A2 eQTL fine-mapped credible set (Supplementary Figures 9). However, the MAPT inversion region remains a highly complex genetic locus to disentangle, partly due to its structural variability and extended linkage disequilibrium.

## Discussion

We sequenced 551 nanopore long-read genomes from healthy older controls and patients with AD and PD to investigate the impact of SVs on neurodegenerative disease. The resulting genomes and SV calls are publicly available, enabling assessment of SV allele frequencies and functional impacts for clinical filtering. Coordinates are provided as a downloadable BED file and through an online browser, allowing identification of variants tolerated in an aging population and prioritization of rare, potentially pathogenic SVs.

Our SV calls are consistent with prior estimates of genome-wide burden, with a median of ~22,000 SVs per genome (Audano et al. 2019; Chaisson et al. 2019). As expected, long-read sequencing enabled the detection of more insertions than short-read methods including repetitive mobile element insertions like Alus. However, we observed more duplications and inversions detected by short-read data, likely due to limitations of alignment-based long-read callers at modest coverage or higher false-positive srGS calls. While assembly-based approaches could improve recovery of complex SVs, they were not feasible here due to sequencing depth constraints. Still, our cohort size was sufficient to saturate the discovery of common variants above 1% allele frequency. That said, the predominantly European ancestry of our cohort limits the generalizability of these allele frequencies, and more lrGS across globally diverse populations is essential to fully capture SV diversity (Billingsley et al. 2024; Gustafson et al. 2024; Mahmoud et al. 2024). While we only performed nanopore-based lrGS, recent benchmarks report high concordance, with ~90% of SVs shared between nanopore and PacBio calls, suggesting that most variants in our dataset would be detected by both technologies, whereas up to 10% may be nanopore-specific (Smolka et al. 2024).

To interpret the potential functional effects of SVs, we leveraged matched transcriptomic and proteomic data from the same individuals. This allowed us to prioritize SVs through both rare variant outlier enrichment and machine learning modeling, as well as common variant QTL mapping. Outlier-based approaches helped validate the regulatory potential of rare variants, discovering a handful of high-impact variants near AD and PD risk genes, while QTL analysis and fine-mapping revealed that SVs may underlie a substantial fraction of known molecular trait associations. At the HP locus, for example, we fine-mapped a 1.7 kb deletion spanning two exons—absent from srGS and poorly tagged by SNVs—as the likely causal variant driving differences in HP protein levels in both plasma and CSF. This previously identified deletion is known to influence HP isoform structure, which affects cholesterol metabolism and has been implicated in AD and PD risk (Bai et al. 2023; Costa-Mallen et al. 2015). This example highlights a broader phenomenon: across our dataset, we observed that many SV-QTLs—especially insertions and mid-sized deletions—were poorly tagged by nearby SNVs and would be missed by traditional GWAS or QTL analyses relying on short-read data. Enabled by lrGS genotyping of these SVs, however, we were able to determine that 12% of fine-mapped credible sets contained an SV, and 6% had an SV as the lead variant. These are likely conservative estimates, as genotyping uncertainty from our relatively low-coverage long-read data likely reduced our power to detect associations.

Our cohort size allowed us to study QTLs down to 2% minor allele frequency, and we found that these low-frequency SVs often had large effect sizes and were less well-tagged by SNVs. These variants may represent an underexplored class of functional regulatory variation with potential relevance to neurodegenerative disease. A key limitation of our proteomics analyses is that the SomaLogic platform used for proteomics may conflate structural disruption of binding sites with true abundance change and only covers a limited set of proteins, so further work to fine-map additional proteins in disease-relevant tissues is needed.

To connect these findings with disease risk, we performed colocalization with AD and PD GWAS data. This nominated several long-read-discovered SVs as candidate causal variants, including *Alu* insertions at *ACE* and *TMEM106B*—both known risk genes—colocalizing with pQTL signals in plasma and CSF. Because long-read sequencing also allows for direct methylation calling, we were able to extend colocalization to methylation QTLs. This revealed potential regulatory SVs near enhancers at genes like *BIN3* that colocalized with GTEx expression of nearby risk genes and Parkinson’s disease GWAS. While our multi-omics profiling was limited to blood expression and CSF/plasma proteomics, we additionally leveraged GTEx QTLs from multiple tissues and SNV–SV LD to prioritize variants likely to act in more disease-relevant tissues. One such example is a large deletion in *NBEAL1*, which colocalizes with multiple neurovascular traits, including AD and vascular dementia and is an eQTL in arterial tissue in GTEx.

In conclusion, by combining long-read genome sequencing with multi-omic functional data, we identified thousands of SVs missed by short-read sequencing and fine-mapped a subset with potential regulatory and disease relevance. Our results highlight the importance of including SVs, particularly those detectable only with long-read technologies, in models of genetic architecture for complex diseases like AD and PD.

## Methods

### Ethics statement

All ADRC and SAMS participants provided written informed consent under Stanford IRB–approved protocols for the use of their data in IRB-approved research.

### Cohorts description

Participants in the Stanford Alzheimer’s Disease Research Center (ADRC) were enrolled through the NIA-funded Stanford ADRC, a longitudinal cohort comprising individuals diagnosed with dementia and cognitively normal elder controls. Diagnostic status was assigned in a consensus conference of board-certified neurologists and neuropsychologists using the Clinical Dementia Rating scale and NACC procedures. Stanford ADRC participants receive neurological exams, comprehensive neuropsychological evaluations, MR and amyloid PET imaging, and donate blood and CSF samples. They are invited back annually for follow-up assessments.

The Stanford Aging and Memory Study (SAMS) is an ongoing NIA-funded longitudinal investigation of healthy aging, enrolling participants who demonstrate no cognitive impairment (Trelle et al. 2021, Sheng et al. 2025). All blood collection, processing protocols, and neuropsychological assessments mirrored those used in the ADRC to ensure methodological consistency. At baseline, each SAMS participant exhibited a Clinical Dementia Rating of 0 and achieved age-adjusted normative scores on a standardized neuropsychological battery; cognitive status was confirmed by the same multidisciplinary consensus panel.

Of note, some participants were enrolled in both ADRC and SAMS.

In the Stanford ADRC and SAMS cohorts, comprehensive biomarker assessments were conducted to evaluate amyloid and tau pathology. Cerebrospinal fluid (CSF) levels of Aβ42, Aβ40, p-tau181, and total tau were measured using Lumipulse G assays, with a CSF Aβ42/Aβ40 ratio below 0.091 indicating amyloid positivity. Plasma p-tau181 and Aβ42/Aβ40 ratios were also assessed using modified Lumipulse assays, demonstrating high concordance with CSF and PET measures. Amyloid PET imaging with 18F-Florbetaben was performed on a subset of participants, and standardized uptake value ratios (SUVRs) were calculated using a global cortical composite relative to the whole cerebellum.

### Defining clinical diagnoses groups

To define neurodegenerative disease groupings of the lrGS sequenced ADRC and SAMS participants, we relied on the comprehensive biomarker data and cognitive assessments as described in a previous study (Winer et al. 2025). In brief, participants underwent diagnostic adjudication at multidisciplinary consensus meetings, which included a panel of neurologists, neuropsychologists, and research staff through their involvement in ADRC or SAMS study. Participants diagnosed clinically with mild cognitive impairment due to AD (MCI) or Alzheimer’s Disease (AD) met criteria for National Institutes of Health Alzheimer’s Disease Diagnostic guidelines and were confirmed to have biomarker evidence of AD using Amyloid-PET and CSF Aβ42/40. Parkinson’s was also diagnosed using the UK Brain Bank criteria and required bradykinesia with muscle rigidity and/or rest tremor. Parkinson’s diagnoses were further split into PD with MCI (PD-MCI) if they had cognitive complaint and objective impairment on cognitive testing, without substantial impact on functional activities, or Parkinson’s disease dementia / Lewy body dementia (PDD/LBD) as cognitive impairment severe enough to interfere with activities of daily living as determined by clinical history and Clinical Dementia Rating.

### Long-read sequencing protocol

Long-read genome sequencing (lrGS) was performed on 575 participants. We obtained genomic DNA from NCRAD whole blood samples or extracted high molecular weight DNA from primary blood mononuclear cells stored at −80 °C using a Puregene kit (Qiagen, Germany). DNA was then sheared using a G-tube (Covaris LLC, Massachusetts). Sequencing libraries were prepared using Nanopore LSK-110 and sequenced on the PromethION48 following standard protocol (Oxford Nanopore Technologies, United Kingdom). We aimed for 60 GB of estimated bases sequenced per sample during sequencing, at which point the run was stopped and the flow cell was washed and then used with the Nanopore WASH-003 kit to be used on an additional sample. We sequenced an average of 1.8 samples per flow cell. After sequencing QC, an average of 50.4 gigabases were sequenced per sample, with a read length N50 of 18 kb. Sequencing data were basecalled using Guppy (High Accuracy, version 6.3) and aligned to hg38 using Minimap2.15. Structural variants were called using Sniffles216 in population mode. Clair3 was used to call SNVs and short indels from lrGS bams and 24 samples were excluded when IBD analysis with srGS variant calls revealed sample swaps or cryptic nonmatching between the lrGS and srGS sequencing.

### Short-read sequencing protocol

Short-read whole-genome sequencing (sWGS) was performed on 588 participants. The majority (n=554) were sequenced using MGI high-throughput sequencing on DNBSEQ-T10 (BaseNum > 90G, Q30 > 85). The rest (n=34) were sequenced as part of the Stanford Extreme Phenotypes in Alzheimer’s Disease project, with sequencing performed at the Uniformed Services University of the Health Sciences (USUHS) on an Illumina HiSeq platform. The Genome Analysis Toolkit (GATK) workflow Germline short variant discovery was used to map genome sequencing data to the reference genome (GRCh38) and to produce high-confidence variant calls using joint-calling (Poplin et al., 2017). Six individuals were excluded from further srGS analysis due to discordance between their reported sex and genetic sex.

### Transcriptomics and proteomics normalization

Single-cell RNA sequencing data were generated for ADRC and SAMS individuals using DNBelab C4 system from MGI, as previously described (Grandke et al. 2025). To map QTLs and call outliers, these single-cell data were aggregated into pseudobulk profiles by summing gene read counts across all barcodes per individual. Library size was estimated from aggregated counts, and for individuals sequenced at multiple time points, we retained the sample with the largest library size. Genes with low variance were excluded using coefficient of variation with the modelGeneCV2 function from scran package and filterByExpr from edgeR leaving 13K genes (Lun, McCarthy, and Marioni 2016; Robinson, McCarthy, and Smyth 2010). Counts were log-transformed, and library-size normalized using edgeR package. The resulting sample-by-gene matrix was then rank-normalized for QTL mapping, and principal component analysis (PCA) was applied to estimate global hidden factors.

For outlier detection, normalized counts were instead scaled and centered to z-scores. PCA was performed on this z-score matrix, and top PCs were regressed out along with age and sex. Residuals were re-scaled to generate final expression z-scores. Individuals with an excessive burden of outlier genes (|z-score| > 3) were removed as global outliers.

Proteomics data were generated using the SomaScan v4.1 platform. Following SomaLogic-recommended normalization, calibration, and QC, raw aptamer intensities were log-transformed and normalized as previously described (Oh et al. 2023). For plasma samples, where most individuals had multiple time points, the sample with the highest connectivity z-score was retained. Low-expression aptamers were removed using edgeR *filterByExpr*, leaving 6.8K aptamers for plasma and 5K for CSF. The normalized matrices were rank-normalized for QTL mapping, and PCA was performed to capture phenotype structure. Outlier detection was performed analogously to RNA: log-transformed protein values were scaled, residualized for age, sex, and top PCs, re-scaled to z-scores, and global outlier samples (excess |z-score| > 3 aptamers) were excluded. SomaScan aptamers were mapped to genomic coordinates by first mapping associated UniProt IDs to corresponding Ensembl gene ids and coordinates using BioMart.

### Methylation data processing and normalization

5mC CpG methylation was called from Oxford Nanopore data using nanopolish/f5c on aligned BAM files (Simpson et al. 2017; Gamaarachchi et al. 2020). Nanopolish methylation calls were summarized with *calculate_methylation_frequency.py*, applying a log-likelihood ratio threshold of 1 to generate per-CpG methylation proportions (beta values) and coverage. These calls were reformatted and indexed for input into METAFORA.

METAFORA aggregated methylation profiles across all samples to generate a tissue mean, then segmented CpG methylation on autosomes into 303K correlated clusters. Per-sample methylation betas were aggregated within segments, producing a sample-by-segment beta matrix. Segments were filtered for variability by transforming betas as *logit(1 – |0.5 – β|)*, which symmetrizes hyper/hypomethylation, mapping values near extremes of 0 and 1 to values close to zero and mapping high-variance segments with mean betas near 0.5 to higher positive values. The coefficient of variation (CV^2^) of transformed values was compared against a loess-estimated mean–variance trend, and 36K segments with residuals >3 standard deviations were defined as variable segments. This subset was rank-normalized for QTL input, and PCA was performed to estimate hidden factors.

Methylation outliers were called with METAFORA using minimum absolute z-score threshold of 2.5, and minimum outlier delta of 0.25. Global outlier samples who had above the tukey outlier limit of total methylation outlier counts per genome were excluded from enrichment analyses.

### QTL mapping

Structural variant (SV) genotypes were obtained from Sniffles2 joint-genotyping VCFs. SVs missing in >50% of individuals were excluded. Minor allele frequencies (MAF) were computed within ADRC, and only common SVs (MAF ≥ 0.02) were retained. Missing genotypes were imputed using missMDA package *imputePCA*, and PCA on the imputed matrix provided genotype PCs, which recapitulated self-reported ancestry.

QTL mapping was performed with *fastLLM* (R), chosen because tensorQTL cannot handle multi-base SV intervals when overlapping cis-windows. A 1 Mb cis-window around each phenotype coordinate (gene TSS, protein-coding gene, or methylation segment) was intersected with SV positions. For each overlap, linear models were fit with phenotype rank-normalized values as response, SV genotype as predictor, and covariates including sex, age, AD/PD diagnosis, and an optimized number of phenotype PCs. The optimal PC number was chosen by maximizing discovery yield (RNA = 30, plasma = 40, CSF = 30, methylation = 7), (Supp. Fig. 4d). From the final model, effect sizes and standard errors were reported. P-values were Bonferroni corrected across tests, and associations with adjusted p < 0.05 were retained.

### SuSiE fine-mapping

Fine-mapping of SV QTLs was performed jointly with short-read SNVs/indels using individual-level SuSiE (Zou et al. 2022). The SV genotype matrix was subset to individuals with matching srGS data. For each phenotype, a 1 Mb cis-window (around TSS for expression/protein, or methylation segment coordinates) was intersected with lrGS SV and srGS SNV/indel calls (via plink). Genotypes were combined, imputed using missMDA *imputePCA*, then scaled and centered. Phenotype residuals (after covariate regression from the SV-QTL model) were likewise scaled.

SuSiE was run with *L = 10* and *min_abs_corr = 0.25*. For each credible set, the variant with highest PIP was designated the lead, and sets were annotated as SV-led or SNV-led. Credible sets were also annotated as containing an SV in credible set even if it was not lead. Enrichment for SVs among credible sets was assessed by applying PIP thresholds and testing enrichment of SVs compared to SNPs using Fisher’s exact test.

### GWAS colocalization

To colocalize ADRC/SAMS QTLs with neurodegenerative GWAS, we obtained summary statistics from Alzheimer’s disease (Bellenguez et al. 2022) and Parkinson’s disease (Nalls et al. 2019; Kim et al. 2024), as well as QTL resources (GTEx eQTLs, deCODE and UKBB plasma pQTLs, and Knight-ADRC CSF pQTLs) (GTEx Consortium 2013; Ferkingstad et al. 2021; Sun et al. 2023; Western et al. 2024). SNP summary statistics were harmonized, sorted, and indexed across each data set. For ADRC/SAMS QTLs, we used SNP beta and p-value summary stats from srGS from the susieR fine-mapping for methylation, RNA, plasma protein, and CSF protein phenotypes.

Significant loci were defined using windows of 100 kb around variants surpassing significance threshold of p < 1e-5 for GWAS data sets and p < 1e-4 for QTL data sets. Overlapping windows containing both a GWAS and QTL signal were tested with *coloc.abf* using p-value-only method (Rasooly, Peloso, and Giambartolomei 2022). We report the posterior probability (PP.H4) of a shared causal variant, retaining colocalizations with PP.H4 > 0.5. Selected loci were visualized using locusCompareR (Liu et al. 2019). For colocalizations with ADRC/SAMS QTL, the colocalizations were annotated based on SV fine-mapping of the corresponding ADRC/SAMS QTL. We highlight colocalizations where the SV was contained in the credible set of the QTL as a proxy for SVs that are also influencing risk in the GWAS.

Risk genes were defined from eQTL and pQTL colocalizations with public QTL resources (GTEx, deCODe, UKBB, Knight-ADRC): for each gene, we retained the maximum PP.H4 across all tissues, and genes with PP.H4 > 0.5 for AD or PD GWAS were included in the prioritized set for rare SV interpretation.

### WatershedSV rare variant annotation collection pipeline

Raw molecular measurements from each omic dataset, including RNA expression, plasma proteomics, and CSF proteomics, were first processed and normalized. To enhance concordance between RNA and protein outliers, RNA reads and protein abundance values were further corrected using principal components: 105 for RNA expression data, and 25 for plasma and CSF proteomics. The data were then scaled and centered, and only genes with an absolute z-score greater than 2 were retained and input into WatershedSV. Z-scores were converted to binary variables to indicate outlier status.

The WatershedSV method was adapted from Jensen et al. (2024) to identify rare SVs with potential functional impact. Whole genome sequencing (WGS) data were processed according to the WatershedSV pipeline previously described. Briefly, rare variants which were annotated with nearby genes within a 100 kb window were identified. These gene-variant pairs were then paired with corresponding genomic annotations, which were used as input for the WatershedSV model, along with outlier data computed above.

WatershedSV was applied separately to generate RNA expression, plasma proteomics, and CSF proteomics models using the dataset from the ADRC cohort. For each model, model weights were learned and stratified by annotation categories, including “Coding”, “Conservation track”, “Encode chromHMM 25 states,” “Noncoding,” “Regulatory,” and “SV”. For each omic layer, the model integrated genomic annotations with molecular outlier status to compute posterior probabilities for each rare SV. Variants with high posterior scores were considered likely to have a functional molecular impact.

The WatershedSV pipeline outputs posterior probabilities ranging from 0 to 1 for each rare SV. Downstream model evaluation involved N2 pair prediction and performance benchmarking against a baseline Genome Annotation Model using the area under the precision-recall curve (AUPRC).

## Supplementary Material

Supplement 1

Supplement 2

## Data Availability

Summary statistics can be downloaded and queried at https://qsushiny.shinyapps.io/adrc_lrs/. Data from specific Stanford cohorts can be requested to the following cohort leaders: ADRC, M.G.D (greicius@stanford.edu) or T.W-C. (twc@stanford.edu); SAMS, E.M. (bmormino@stanford.edu) or A.D.W. (awagner@stanford.edu).
