# Supplementary material for: Long-read genome sequencing and multi-omics in aging and neurodegeneration
Source: medRxiv. 2025 Oct 29:2025.10.10.25337775. Preprint. [Version 3] doi: 10.1101/2025.10.10.25337775 (PMC12633103; doi:10.1101/2025.10.10.25337775)
Supplement: Supplement 1 [file media-1.pdf]

# Long-read genome sequencing and multi-omics in aging and neurodegeneration

# Extended Data Tables

## Extended Data Table 1. Demographics long-read sequencing cohort by diagnosis group

| Latest diagnosis groups: | HC | AD | MCI | PD.MCI | PD | LBD/PDD | Other |
|---|---|---|---|---|---|---|---|
| **N lrGS** | 300 (54.4%) | 67 (12.2%) | 79 (14.3%) | 33 (6.0%) | 39 (7.1%) | 23 (4.2%) | 10 (1.8%) |
| Age at collection, mean (SD) | 71.1 (7.6) | 72.4 (10.8) | 75.8 (7.2) | 69.6 (9.2) | 69.2 (6.5) | 73.6 (6.1) | 76.5 (8.9) |
| Age at latest diagnosis, mean (SD) | 72.9 (8.4) | 74.3 (11.0) | 77.7 (7.7) | 72.5 (9.0) | 72.1 (6.6) | 75.2 (6.4) | 79.0 (9.5) |
| Sex, female n (%) | 176 (58.7%) | 35 (52.2%) | 36 (45.6%) | 11 (33.3%) | 19 (48.7%) | 6 (26.1%) | 6 (60.0%) |
| **Race** | | | | | | | |
| White | 257 (85.7%) | 50 (74.6%) | 59 (74.7%) | 32 (97.0%) | 39 (100.0%) | 23 (100.0%) | 5 (50.0%) |
| Asian | 22 (7.3%) | 4 (6.0%) | 8 (10.1%) | 1 (3.0%) | 0 (0.0%) | 0 (0.0%) | 1 (10.0%) |
| Black or African American | 2 (0.7%) | 2 (3.0%) | 2 (2.5%) | 0 (0.0%) | 0 (0.0%) | 0 (0.0%) | 0 (0.0%) |
| More Than One Race | 4 (1.3%) | 0 (0.0%) | 0 (0.0%) | 0 (0.0%) | 0 (0.0%) | 0 (0.0%) | 0 (0.0%) |
| American Indian / Alaska Native | 1 (0.3%) | 1 (1.5%) | 0 (0.0%) | 0 (0.0%) | 0 (0.0%) | 0 (0.0%) | 0 (0.0%) |
| Native Hawaiian or Pacific Islander | 0 (0.0%) | 1 (1.5%) | 1 (1.3%) | 0 (0.0%) | 0 (0.0%) | 0 (0.0%) | 0 (0.0%) |
| **Ethnicity** | | | | | | | |
| Hispanic or Latino | 29 (9.7%) | 8 (11.9%) | 18 (22.8%) | 3 (9.1%) | 2 (5.1%) | 1 (4.3%) | 2 (20.0%) |
| ***APOE* genotype** | | | | | | | |
| 33 | 196 (65.3%) | 18 (26.9%) | 36 (45.6%) | 23 (69.7%) | 24 (61.5%) | 10 (43.5%) | 4 (40.0%) |
| 34 | 59 (19.7%) | 34 (50.7%) | 29 (36.7%) | 6 (18.2%) | 9 (23.1%) | 12 (52.2%) | 2 (20.0%) |
| 23 | 24 (8.0%) | 2 (3.0%) | 8 (10.1%) | 3 (9.1%) | 6 (15.4%) | 1 (4.3%) | 2 (20.0%) |
| 44 | 16 (5.3%) | 12 (17.9%) | 5 (6.3%) | 1 (3.0%) | 0 (0.0%) | 0 (0.0%) | 1 (10.0%) |
| 24 | 4 (1.3%) | 1 (1.5%) | 1 (1.3%) | 0 (0.0%) | 0 (0.0%) | 0 (0.0%) | 1 (10.0%) |
| 22 | 1 (0.3%) | 0 (0.0%) | 0 (0.0%) | 0 (0.0%) | 0 (0.0%) | 0 (0.0%) | 0 (0.0%) |
| **srGS N (%)** | 240 (80.0%) | 49 (73.1%) | 60 (75.9%) | 31 (93.9%) | 32 (82.1%) | 21 (91.3%) | 4 (40.0%) |
| **Plasma proteomics N (%)** | 275 (91.7%) | 55 (82.1%) | 63 (79.7%) | 32 (97.0%) | 37 (94.9%) | 21 (91.3%) | 7 (70.0%) |
| **Blood single-cell RNA seq N (%)** | 132 (44.0%) | 42 (62.7%) | 54 (68.4%) | 27 (81.8%) | 31 (79.5%) | 18 (78.3%) | 4 (40.0%) |
| **CSF proteomics N (%)** | 20 (6.7%) | 9 (13.4%) | 3 (3.8%) | 3 (9.1%) | 8 (20.5%) | 1 (4.3%) | 1 (10.0%) |

## Extended Data Table 2. GWAS risk loci colocalized with SV-finemapped QTLs

| GWAS | locus name / gene | GWAS Lead SNP(s) | Coloc count | mQTL Colocs | eQTL Colocs | pQTL Colocs | Finemapped SV(s) |
|---|---|---|---|---|---|---|---|
| Alzheimer's Disease (Bellenguez et al 2022) | **IDUA** | chr4:772737, chr4:877887, chr4:993555 | 3 | | | **IDUA**.3169.70.2 in plasma (3) | 201bp tandem repeat insertion in 3' UTR of **FGFRL1** |
| | **SERPINB1** | chr6:2848725 | 1 | | | **SERPINB1**.10737.96.3 in CSF (1) | 31bp intergenic tandem repeat indel |
| | **TMEM106B** | chr7:12229042 | 1 | | | **TMEM106B**.8687.26.3 in plasma (1) | 322bp deletion of reference *Alu* element in 3' UTR of **TMEM106B** |
| | **SPI1** | chr11:47387500 | 1 | chr11:48256675-48258636 (1) | | | 101bp intergenic tandem repeat insertion in NR2F2 ChIP-seq peak |
| | **GGA2 / COG7** | chr16:23466676 | 1 | chr16:23504004-23505148 (1) | | | 208bp tandem repeat deletion near **COG7** promoter |
| | **LLGL1** | chr17:18141348, chr17:18249987 | 2 | | **LLGL1** (2) | | 50bp tandem repeat insertion intronic to **EVPLL** |
| | **MAPT / KANSL1 / LRRC37A2** | chr17:46107462, chr17:46212466, chr17:46613342, chr17:46720553 | 9 | chr17:45770939-45772084 (3) | **LRCC37A2** (2), **LRRC37A17P** (4) | | 38bp tandem repeat indel in intron 1 of **MAPT** *lead varaint* for **LRCC37A2** eQTL; 323bp deletion of reference *Alu* element in intron of **KANSL1** finemapped for mQTL region and **LRRC37A17P** eQTL |
| | **ACE** | chr17:63476980 | 1 | | | **ACE**.10714.7.3 in CSF | 291bp *Alu* insertion intronic to **ACE** |
| Parkinson's Disease (Kim et al 2023 ) | **GPNMB** | chr7:23076996, chr7:23231412, chr7:23350848 | 3 | | | **GPNMB**.8289.8.3 in plasma (3) | 30bp intergenic deletion proximal to CTCF binding site and distal enhancer |
| | **BIN3 / CCAR2** | chr8:22613886 | 1 | chr8:22626148-22627181 (1) | | | 329bp insertion of *Alu* element intronic to **BIN3** proximal to enhancer |
| | **NCOR1 / PIGL** | chr17:15964977, chr17:16067368, chr17:16184155, chr17:16306241 | 4 | chr17:16216217-16216772 (7) | | | 32bp deletion in WDR5 ChIP-seq peak intronic to **ZSWIM7** *lead variant* for mQTL of **NCOR1 / PIGL** bidirectional promoter |
| | **MAPT / KANSL1 / LRRC37A2** | chr17:45198582, chr17:45639765, chr17:45756458, chr17:45935097, chr17:46038074, chr17:46169798, chr17:46276519 | 17 | chr17:45592253-45593386 (2), chr17:45770939-45772084 (7), chr17:45813112-45813885 (1) | **LRRC37A2** (2), **LRRC37A4P** (1), **LRRC37A17P** (4) | | 38bp tandem repeat indel in intron 1 of **MAPT** *lead varaint* for **LRCC37A2** eQTL; 323bp deletion of reference *Alu* element in intron of **KANSL1** finemapped for mQTL regions and **LRRC37A17P** eQTL, and others |

# Extended Data and Supplemental Figures

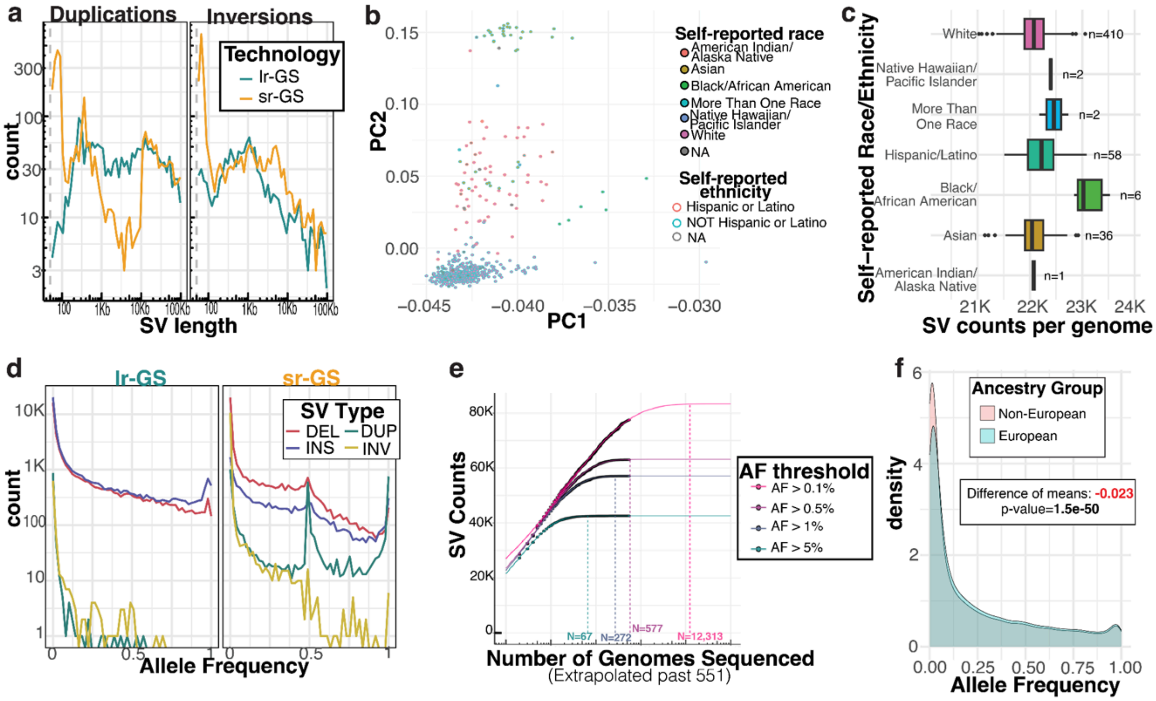


**Extended Data Figure 1. a,** count of duplications and inversions in 366 matched lrGS and srGS samples stratified by length **b,** principal component of common variant SV genotypes showing population stratification (MAF<5%) colored by self-reported race and ethnicity **c,** counts of SVs per genome stratified by self-reported race and ethnicity, count of individuals in each race/ethnicity category annotated **d,** count of SVs across frequency spectrum in lrGS compared to srGS **e,** counts of SVs above various MAF thresholds detected as a function of number of genomes sequenced. Counts for each MAF threshold were fit to a Weibull growth model to estimate the asymptote, and the model was used to extrapolate beyond ADRC genomes and find saturation points defined as the number of genomes needed to attain 99.9% the value of the predicted asymptote. **f,** allele frequency distribution of variants detected in 100 European versus non-European participants in the ADRC/SAMS cohort, showing AF distribution shifted to the left for non-European individuals. T-test estimate and p-value annotated.

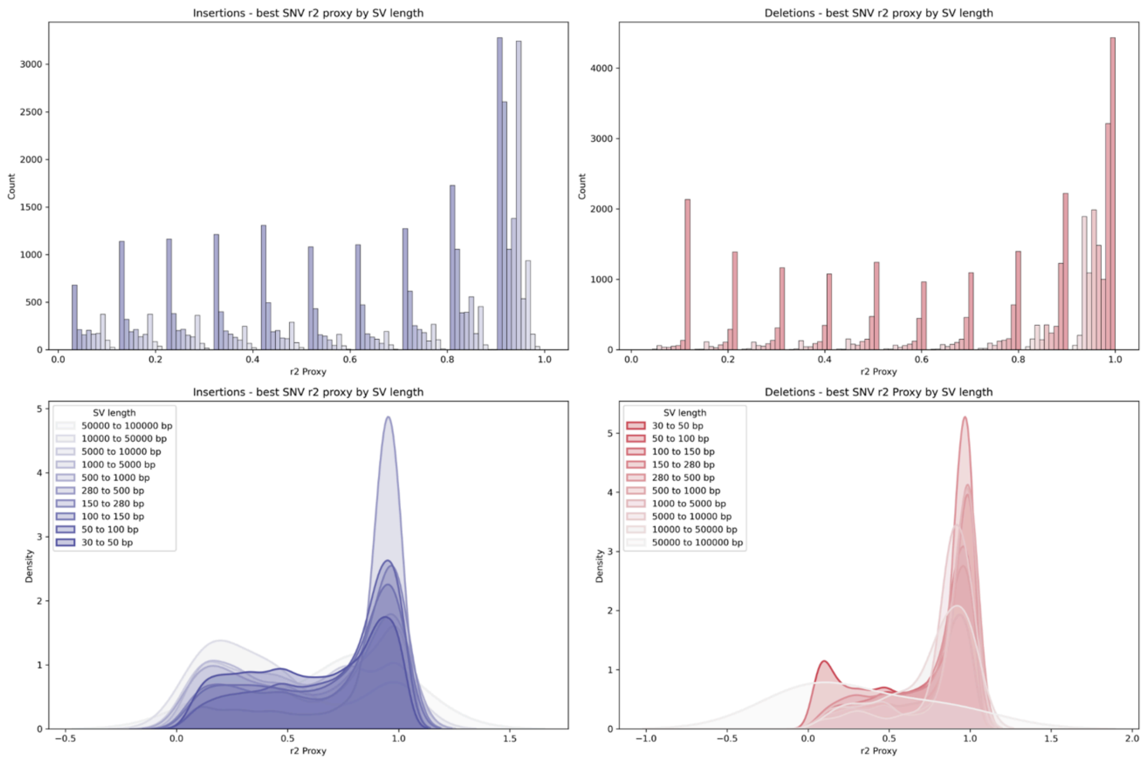


**Extended Data Figure 2.** Distribution of maximum linkage disequilibrium ($r^2$) between common structural variants (MAF >2%) and nearby single-nucleotide variants (SNVs) within 1 Mb, stratified by SV type (insertions vs. deletions) and length. Short SVs (<150 bp), particularly insertions, exhibit low $r^2$ values, indicating poor tagging by adjacent SNVs, while mid-sized SVs (150–1000 bp) show improved tagging efficiency.

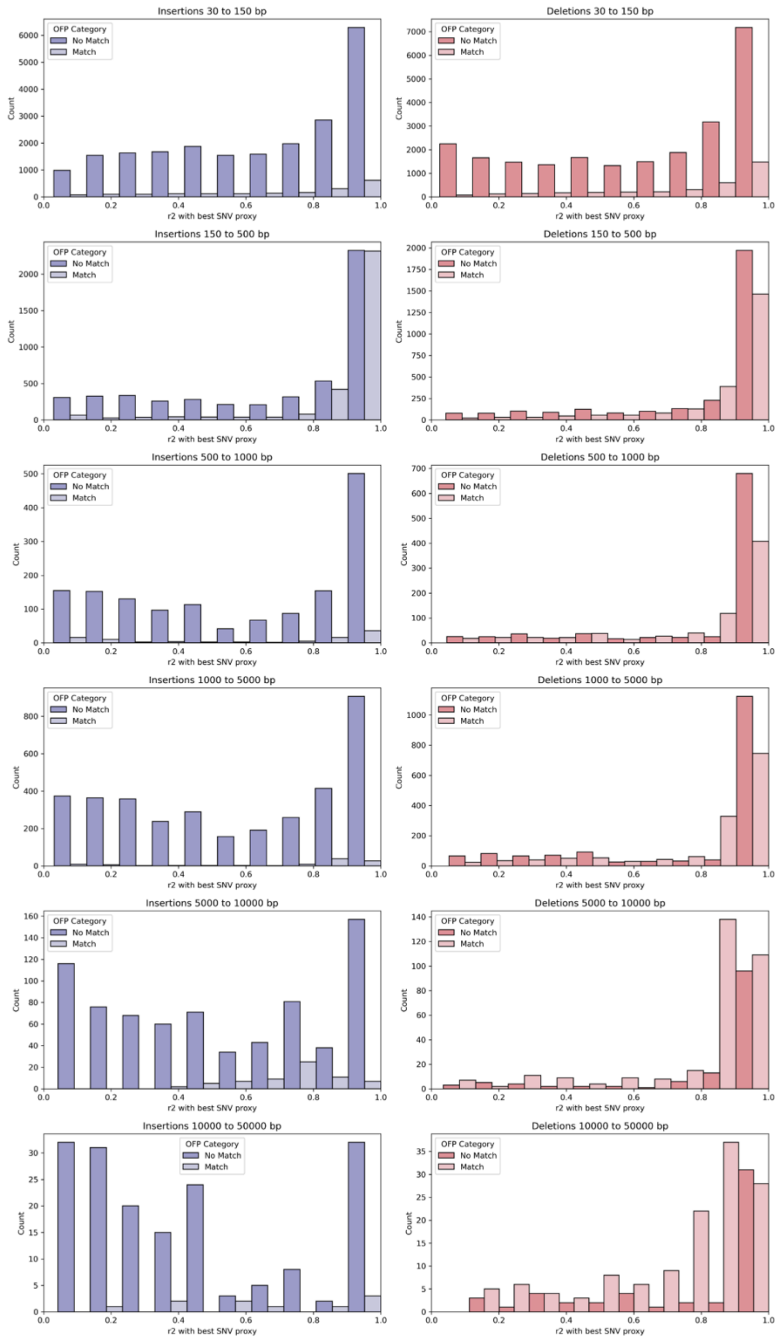


**Extended Data Figure 3.** Relationship between SV overlap with gnomAD v4 short-read SVs (overlap fraction, OFP) and maximum r² with nearby SNVs, stratified by SV length. SVs with higher OFP values tend to have stronger LD with adjacent SNVs, with this trend being more pronounced in mid-sized insertions and deletions.

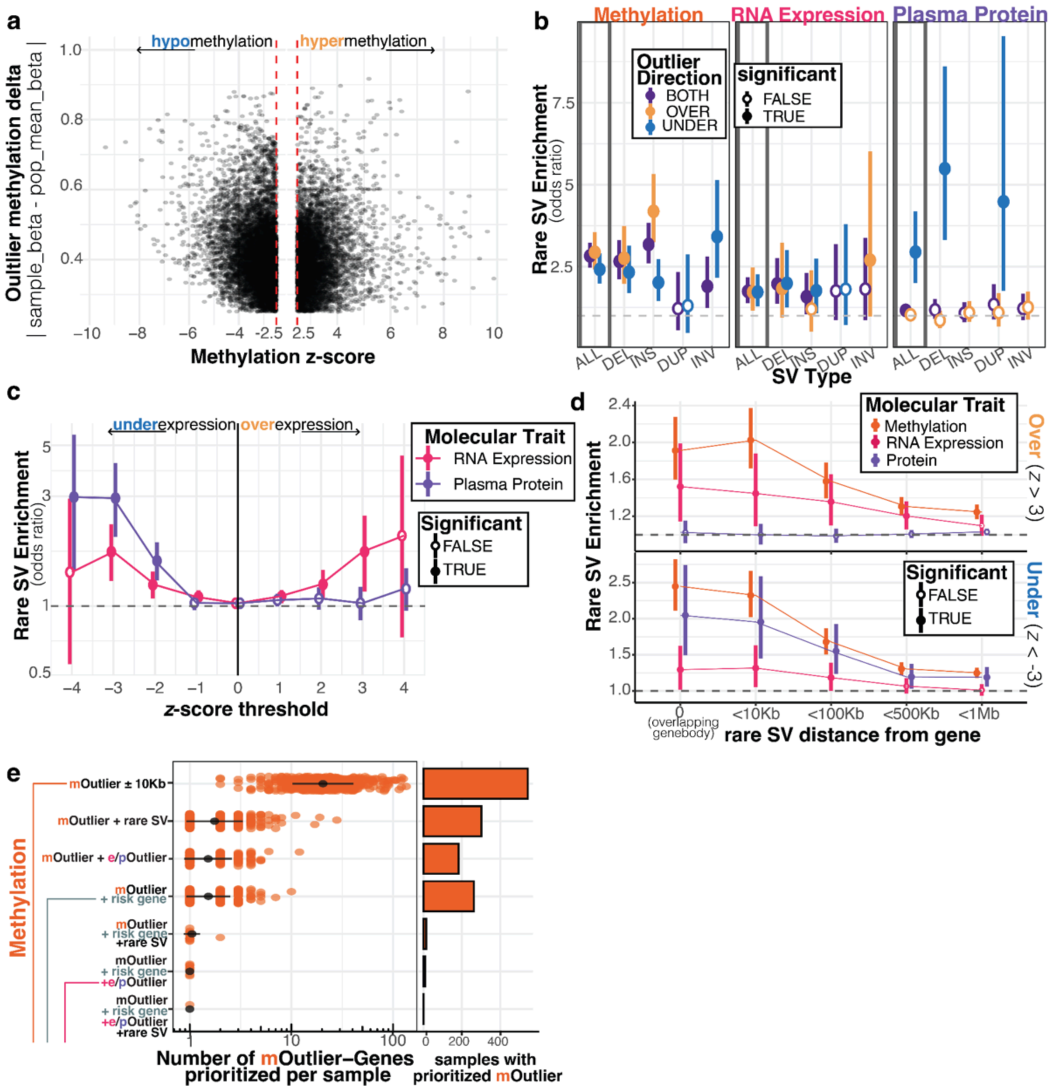


**Extended Data Figure 4. a,** Methylation outliers called from METAFORA stratified by z-score and methylation delta. Methylation delta is the absolute difference between sample's mean proportion of methylated CpGs to the median prop of methylated CpGs across all samples over that outlier region. **b,** SV-specific enrichment of rare variants near outliers (z-threshold=3) across 3 different molecular traits. Values not reported for examples where fewer than 5 rare variants were observed near outliers. **c,** rare variant enrichment for RNA and protein outliers as a function of z-score threshold used to define outliers. Position z-score threshold for over-expression (outlier = z > z_threshold), negative z-scores for under-expression (outlier = z < z_threshold). **d,** rare variant enrichment for outliers across molecular traits as a function of distance to the outlier gene. Distance of 0 corresponds to SV overlapping the gene body. **e** Prioritization of methylation outlier regions per sample. A dot plot displays the number of methylation outliers detected per sample, matching corresponding filters on the y-axis. Bar plot displays the number of samples with at least one methylation outlier meeting those filters.

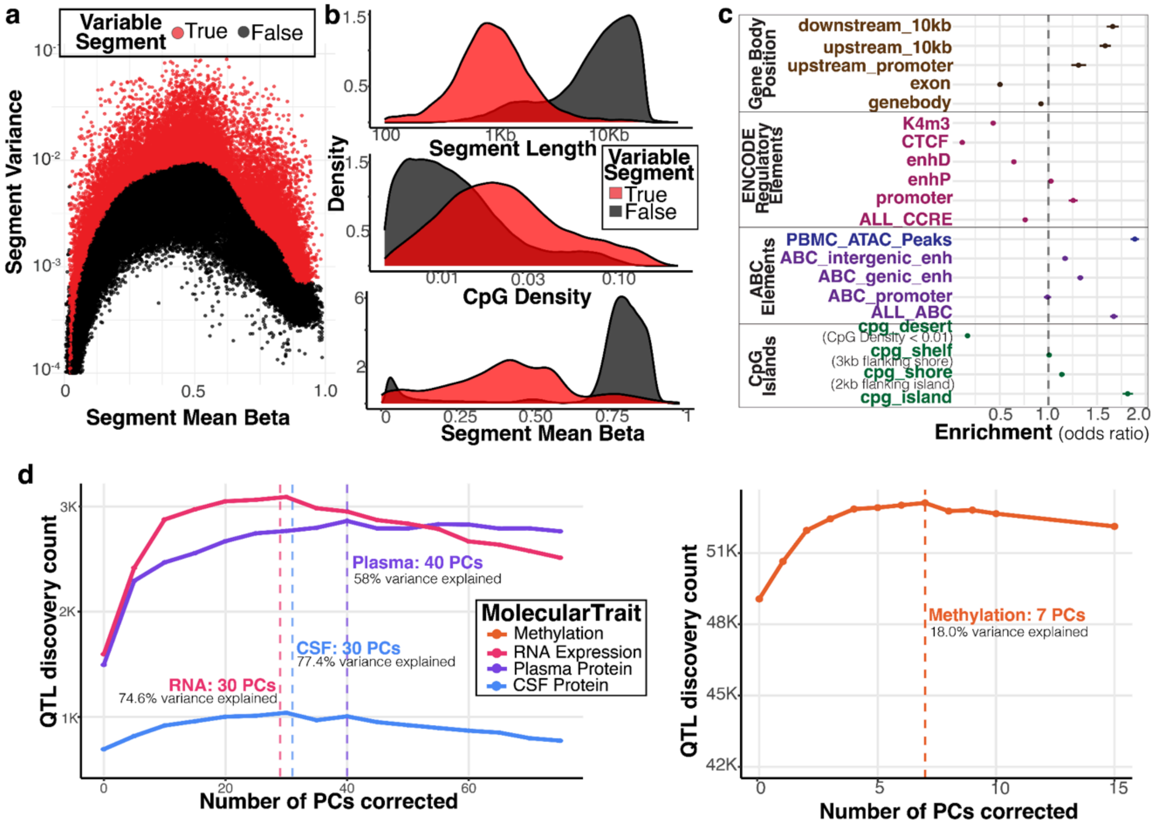


**Extended Data Figure 5. a,** mean methylation betas and variance of 360K methylation regions detected from segmenting population mean methylation beta profile. Segments in red we define as variable after modeling the mean-variance relationship across segments. Variable segments have variance above 3 standard errors of predicted variance, given that segments' mean beta. **b,** distribution of variable methylation segments compared to background segments across 3 different metrics, length of segment (base pairs), CpG density (number of cpg sites per 100 bases), segment mean beta (mean proportion of methylated bases across all CpGs in segment). **c,** enrichment of various genome annotations pulled from ENCODE, ABC models, UCSC cpg island track, and gencode v32 gene coordinates for overlapping variable segments. **d,** number of QTLs detected at an FDR threshold of 5% for a variety of QTL models correcting for an increasing number of phenotype PCs across the 4 molecular traits. The number of PCs achieving a maximum of QTL discoveries is shown with a vertical line, and the percent of variance explained by those PCs is also annotated.

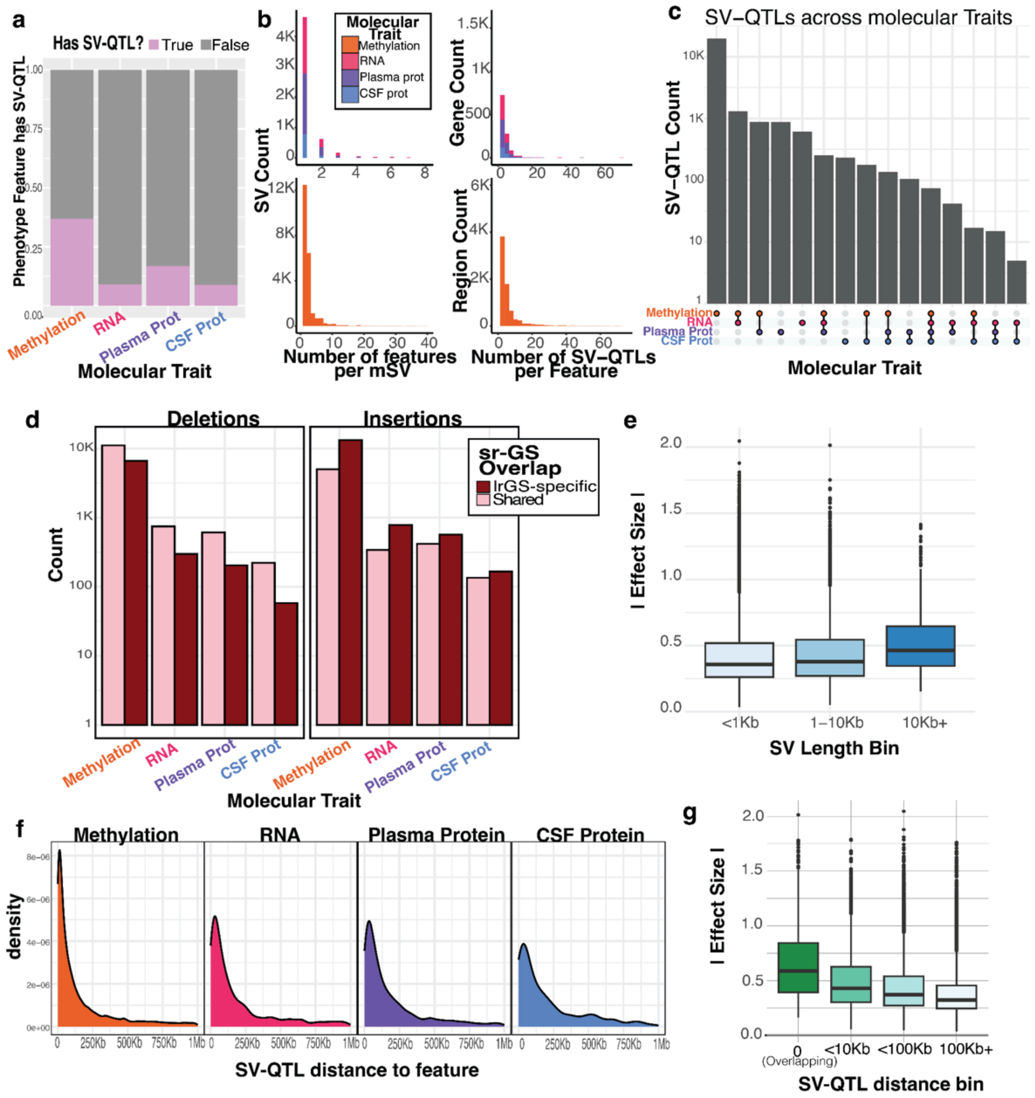


**Extended Data Figure 6. a,** percent of molecular trait features (genes for expression, SomaLogic aptamers for plasma/csf protein, and variable methylation segments for Methylation) with a detected SV-QTL. **b**, histogram showing the distribution of both the number of features associated with each SV and the number of SVs associated with each feature. Methylation is plotted on a separate axis for clarity, as it has higher counts. **c**, upset plot of the sharing of QTLs across molecular traits for eSVs. Each unique SVID was overlapped across all significant SV-QTLs for each molecular trait. **d**, count of SV-QTLs where the SV was uniquely detected by lr-GS, or also present in sr-GS-based population resources like gnomad v4. **e**, absolute effect size distribution of SV-QTLs in different length bins shows that larger variants have a higher effect. **f**, distribution of SV-QTLs based on the distance to their target feature (TSS for RNA and protein, and methylation region for Methylation). Methylation had significantly closer associations compared to other omes. **g**, absolute effect size distribution of SV-QTLs binned by how close they are to the target feature. 0 represents an SV that directly overlapped the target feature

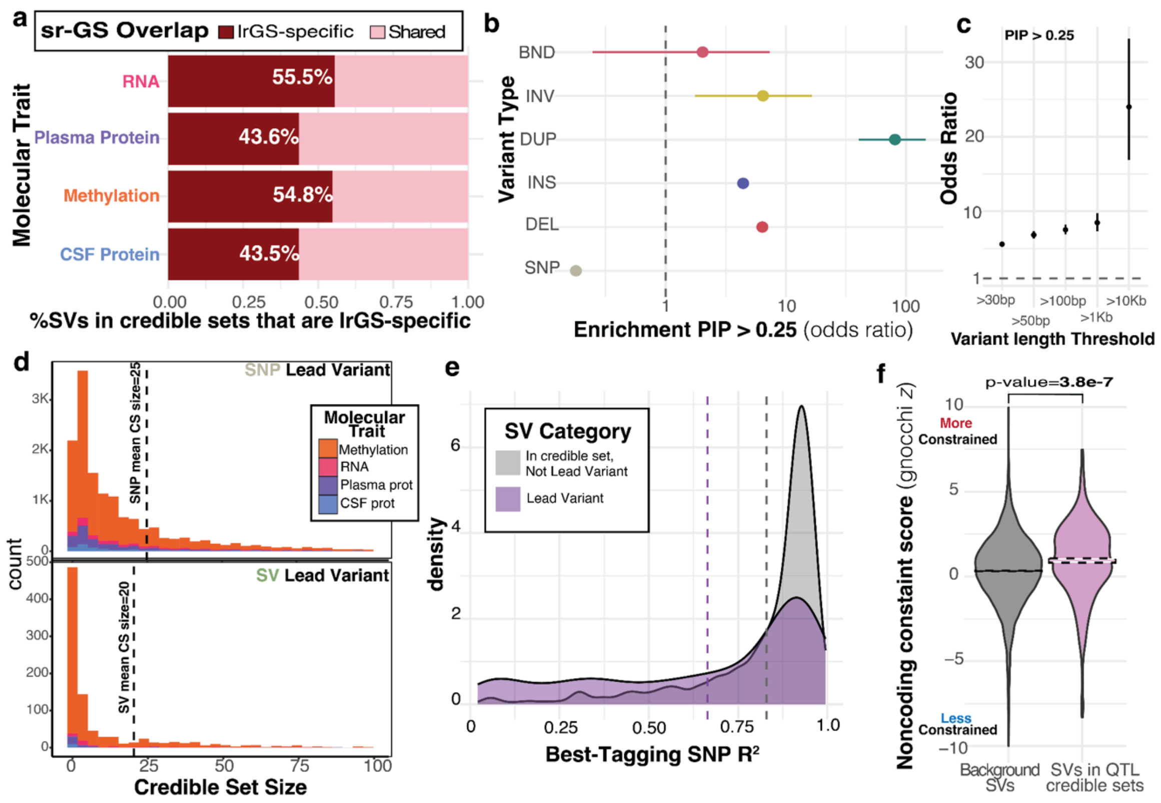


**Extended Data Figure 7. a,** percent of SVs contained in QTL credible sets that were lrGS-specific (absent from sr-GS based population references) **b,** enrichment across variant types for being fine-mapped with PIP above 0.25. Odds ratio from fisher's exact test plotted on x-axis **c,** enrichment for variants above various length thresholds being fine-mapped with PIP above 0.25 **d**, number of variants included in credible sets stratified by whether lead variant of credible set (highest pip variant) was a SNP or SV **e,** distribution of the LD of the best-tagging SNP for lead SVs compared to background SV-QTLs **f,** distribution of max non-coding constraint score (gnocchi score) for regions overlapping SV, comparing fine-mapped SVs to all background SVs.

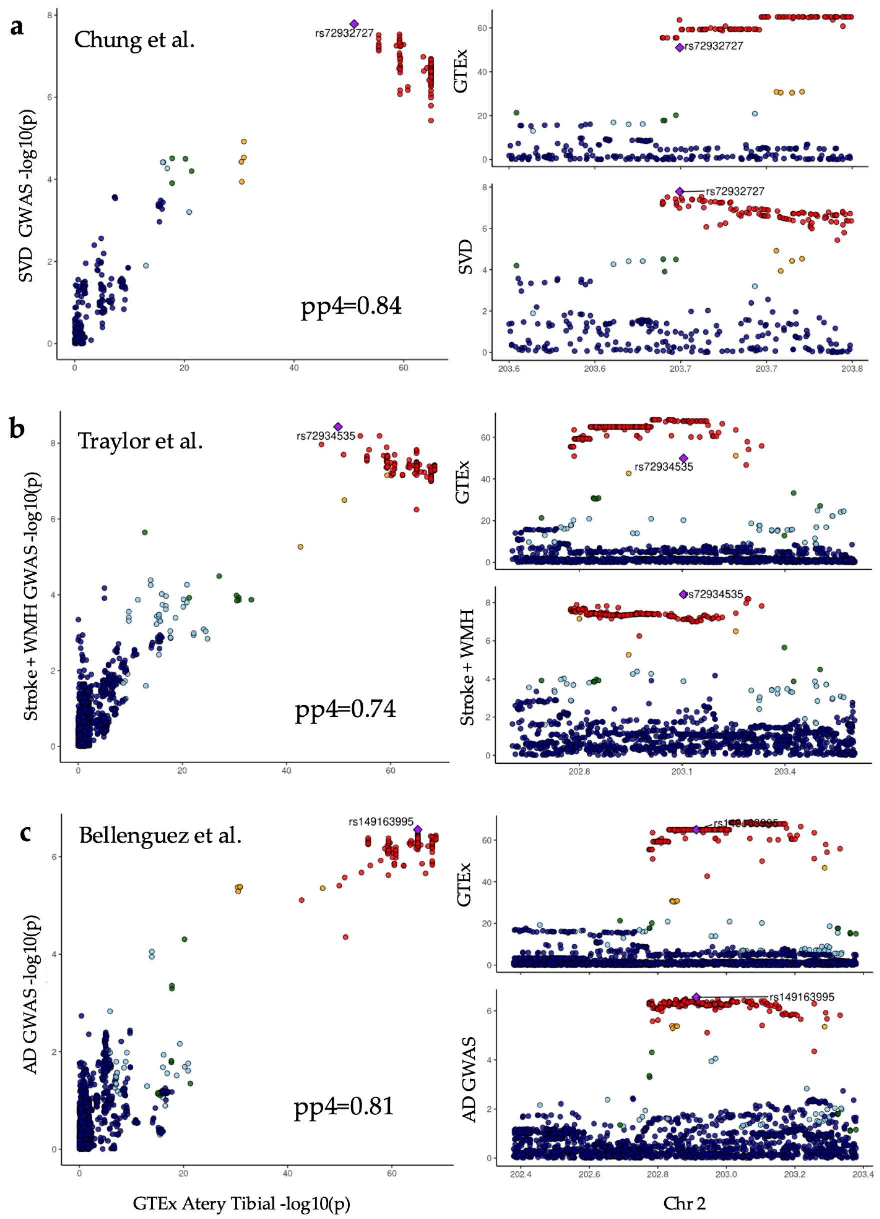


**Extended Data Figure 8. Colocalization of *NBEAL1* expression in GTEx Artery Tibial with Alzheimer's disease and neurovascular GWAS loci**. Colocalization plots showing *NBEAL1* expression in GTEx Artery Tibial and GWAS signals for: **a,)** Small vessel disease (SVD) (Chung et al., 2022 PMID: 31430377), **b,** Stroke and white matter hyperintensities (WMH) (Traylor et al., 2021, PMID: 33773637), **c,** Alzheimer's disease (AD) (Bellenguez et al., 2024,PMID: 35379992). This locus includes a large intronic deletion in *NBEAL1* (chr2:203034349–203039584; 5,235 bp), in high LD with SNPs previously associated with AD (rs139643391, *WDR12*, $R^2$=0.92), SVD (rs72932727, *ICA1L*, $R^2$=0.89), ischemic stroke, and WMH (rs72934535, *NBEAL1*, $R^2$=0.82). Posterior probabilities of colocalization (pp4)

are shown: SVD (PP4 = 0.83), WMH (PP4 = 0.74), and AD (PP4 = 0.81). Lead GWAS SNPs are labeled in the GTEx and GWAS SNPs intersection.

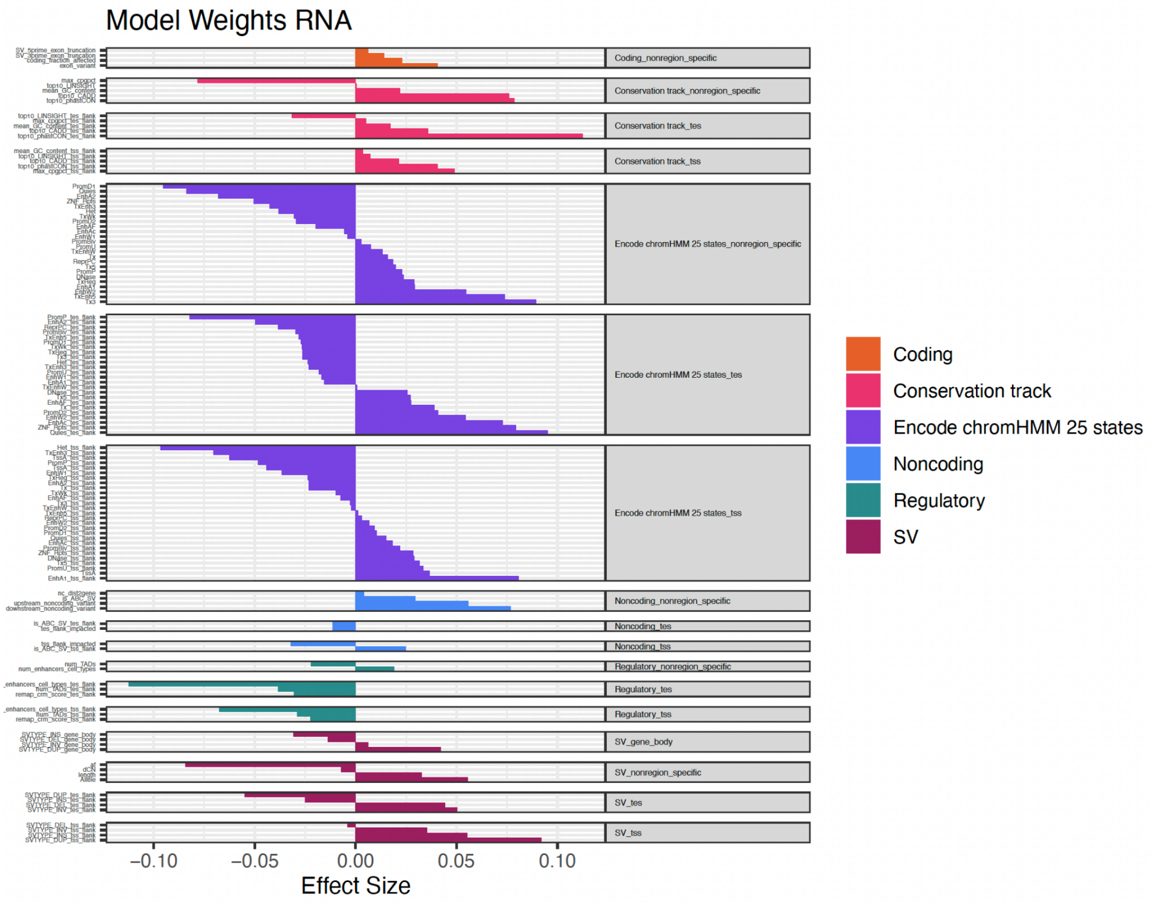


**Supplementary Figure 1.** Watershed model weights for RNA expression data were stratified by both genomic regions (including gene body, transcription start site (TSS), and transcription end site (TES)) and gene annotation categories. These categories included: coding (non-region specific), noncoding (non-region specific), regulatory (non-region specific), conservation track (non-region specific), ENCODE ChromHMM25 states (non-region specific), and structural variants (SVs) located within the gene body.

The strongest expression-related annotations were: (1) structural variants (SVs) located within exon regions, (2) SVs in proximity to genes that intersect an ABC enhancer assigned to the corresponding gene, and (3) the number of primary tissues in which an SV within an enhancer BED segment is observed.

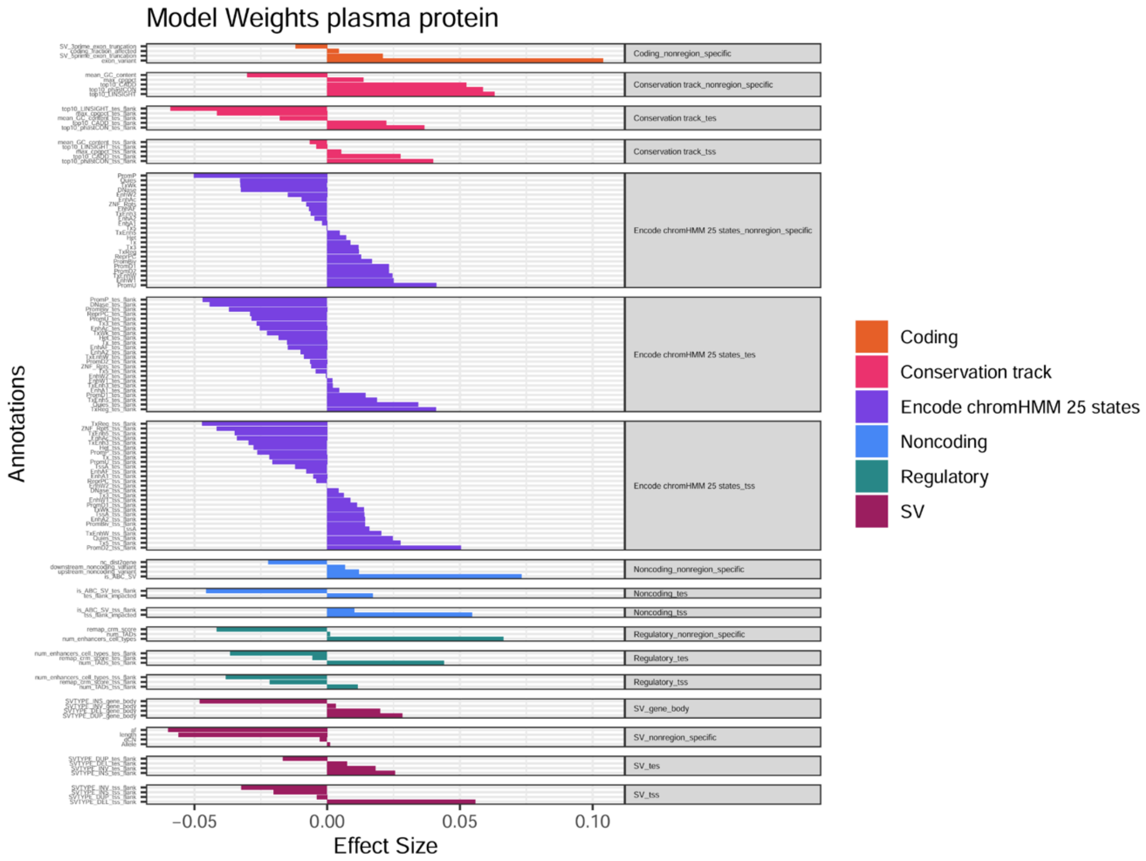


**Supplementary Figure 2.** For plasma protein, the top three annotation levels included: (1) SVs situated in exon regions, (2) SVs near genes overlapping with ABC enhancers linked to the gene, and (3) the mean of the top 10 LINSIGHT conservation scores across the SV span.

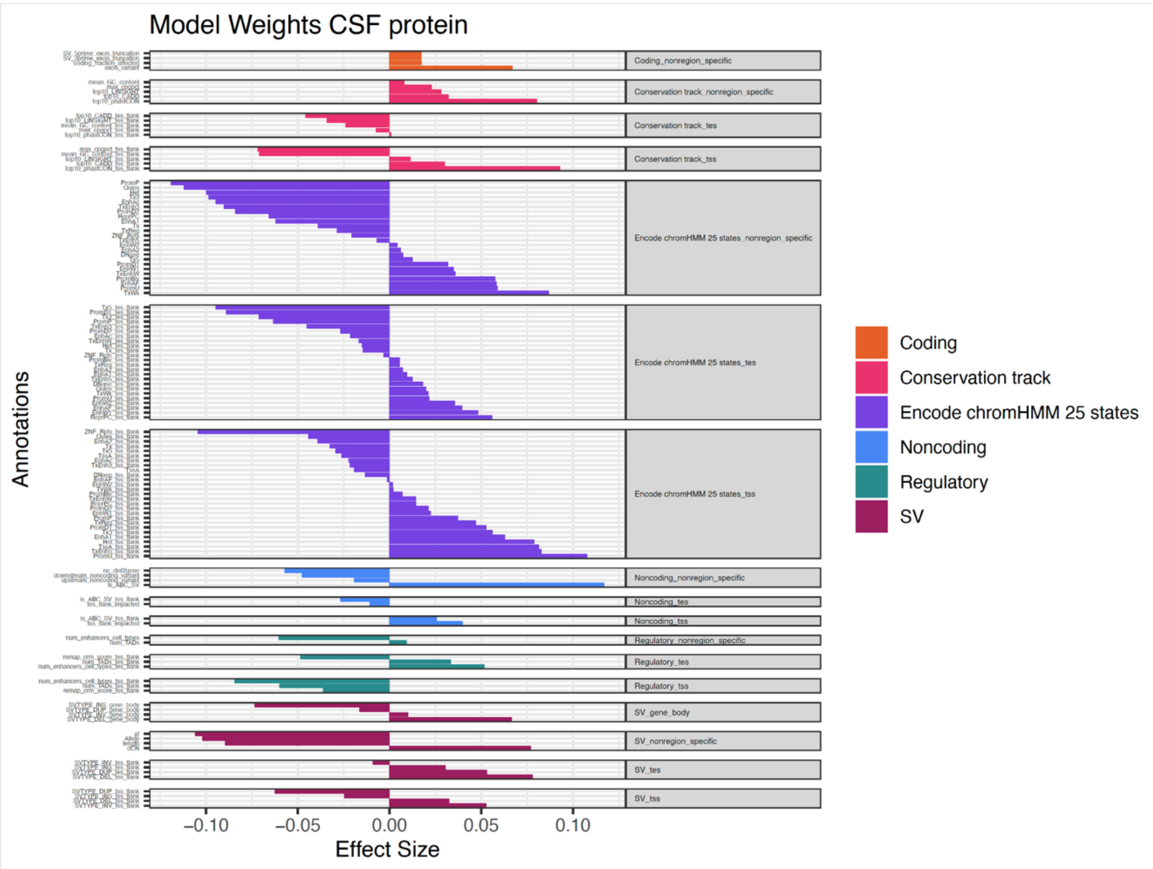


**Supplementary Figure 3.** The top annotations for CSF protein levels were: (1) SVs near genes that overlap ABC enhancers mapped to the gene, (2) SVs in the upstream promoter-flanking region of a transcription start site (TSS), and (3) the average of the top 10 phastCons conservation scores across the SV region.

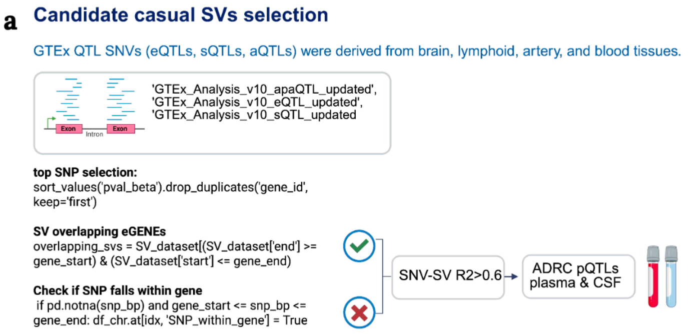


**b**

| | Count |
|---|---|
| Top SNPs | 294,118 |
| apaQTL | 25,904 |
| eQTL | 208,776 |
| sQTL | 59,438 |
| SNPs in eGENE | 74,595 |
| SNPs not in eGENE * | 219,523 |
| eGENE has CSF aptamer - ADRC | 61,498 |
| eGENE has Plasma aptamer - ADRC | 84,033 |
| At least one SV in eGENE * | 219,917 |
| SNP-SV pairs (LD R2>0.6)* | 805 |
| SNP-SV pairs with CSF aptamer | 319 |
| SNP-SV pairs with Plasma aptamer | 493 |
| Significant Multiple Regression Model pQTL CSF** | 65 |
| Significant Multiple Regression Model pQTL Plasma** | 129 |
| AIC SV model < SNP CSF | 29 |
| AIC SV model < SNP Plasma | 62 |

**Supplementary Figure 4.** Identification of candidate causal SVs through GTEx QTL colocalization and ADRC proteomic prediction. **a,** Schematic workflow for selecting structural variants (SVs) potentially involved in gene regulation. GTEx QTL datasets—eQTLs (expression QTLs), sQTLs (splicing QTLs), and aQTLs (alternative polyadenylation QTLs)—were derived from brain, lymphoid, artery, and blood tissues. For each QTL type, the top SNP per gene was selected by sorting on p-values. SVs overlapping eGENEs were retained based on genomic coordinates. SNP–SV pairs in high linkage disequilibrium (LD $R^2 > 0.6$) were then tested in the ADRC cohort for associations with CSF and plasma protein levels (pQTLs), specifically when the top SNP did not fall within the eGENE body. **b,** Summary of the number of SNPs, QTLs, and SV–gene overlaps at each step of the pipeline. Counts include eGENEs with matching CSF or plasma aptamers in ADRC, and SNP–SV pairs with LD $R^2 > 0.6$. Asterisk (*) indicates LD calculation between SNP and SV. Double asterisk (**) indicates that either the SV or SNP showed significance in a multiple regression model (p-value < 0.05). Model fit was evaluated using Akaike Information Criterion (AIC), with a lower AIC indicating better model performance.

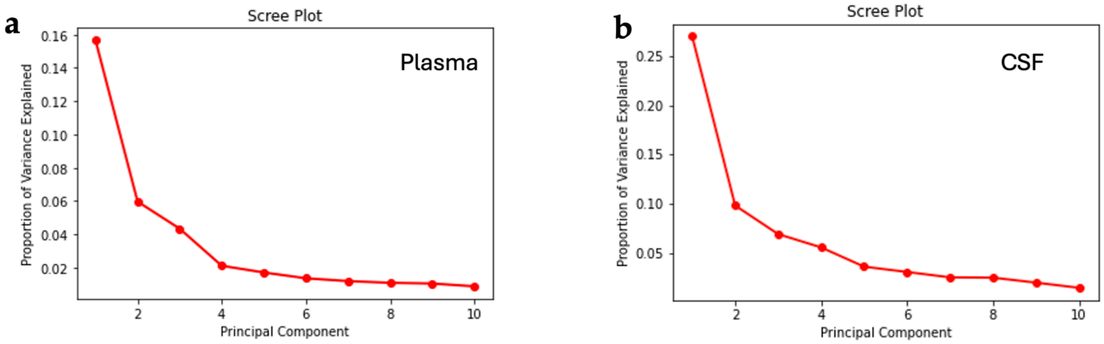


**Supplementary Figure 5.** Principal components of ADRC proteomics for GTEx loci multiple regression models. a, Elbow plots showing the principal components derived from plasma proteomics and b, cerebrospinal fluid (CSF) in the ADRC cohort. For the eGENE–proteomics analyses, four principal components were included in the plasma models and six in the CSF models.

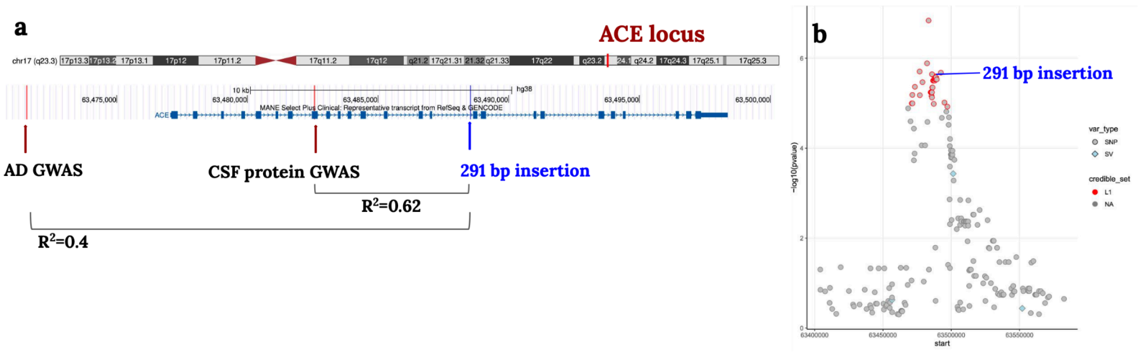


**Supplementary Figure 6.** Fine-mapping of the *ACE* Locus for ADRC CSF Proteomics. **a,** Genomic view of the *ACE* locus showing the AD GWAS SNP (rs4277405, Bellenguez et al., 2024), the CSF protein GWAS SNP (rs4309, Cruchaga et al., 2023), and a 291 bp Alu insertion (chr17:63488532). Linkage disequilibrium values ($R^2$) are indicated. **b,** Fine-mapping in the ADRC cohort for *ACE* CSF proteomics (*ACE.10714.7.3.ENSG00000159640.ACE* SomaScan aptamer): –log10(p-values) plotted by genomic position. SNPs are shown as circles, SVs as diamonds. Variants in the L1 credible set are highlighted in red, including the 291 bp insertion.

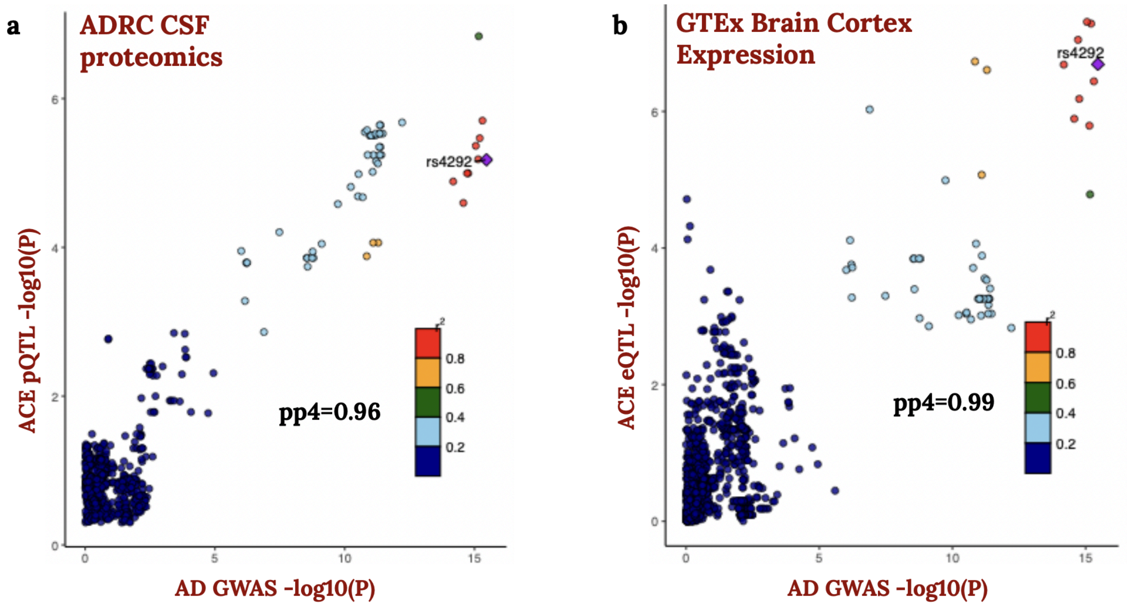


**Supplementary Figure 7.** Colocalization of *ACE* molecular traits with Alzheimer's disease (AD) GWAS in the ADRC and GTEx dataset. Colocalization plots between AD GWAS (Bellenguez et al., 2024, PMID: 35379992):**a,** *ACE* CSF protein levels in the ADRC cohort (pQTL); **b,** *ACE* expression in GTEx Brain Cortex (eQTL). Posterior probabilities (pp4) of colocalization are shown. Lead GWAS SNPs are labeled in the GTEx and GWAS SNPs intersection.

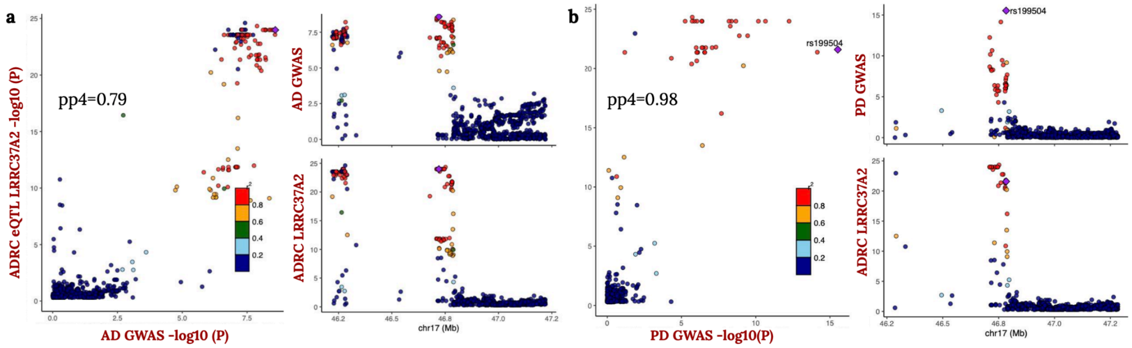


**Supplementary Figure 8.** Colocalization of ADRC *LRRC37A2* eQTL with AD and PD GWAS signals. **a,** Colocalization between the *LRRC37A2* eQTL (ENSG00000238083) from the ADRC cohort and Alzheimer's disease (AD) GWAS (Bellenguez et al., 2024). **b,** Colocalization between the same ADRC *LRRC37A2* eQTL (ENSG00000238083) and Parkinson’s disease (PD) GWAS (Kim et al., 2024).

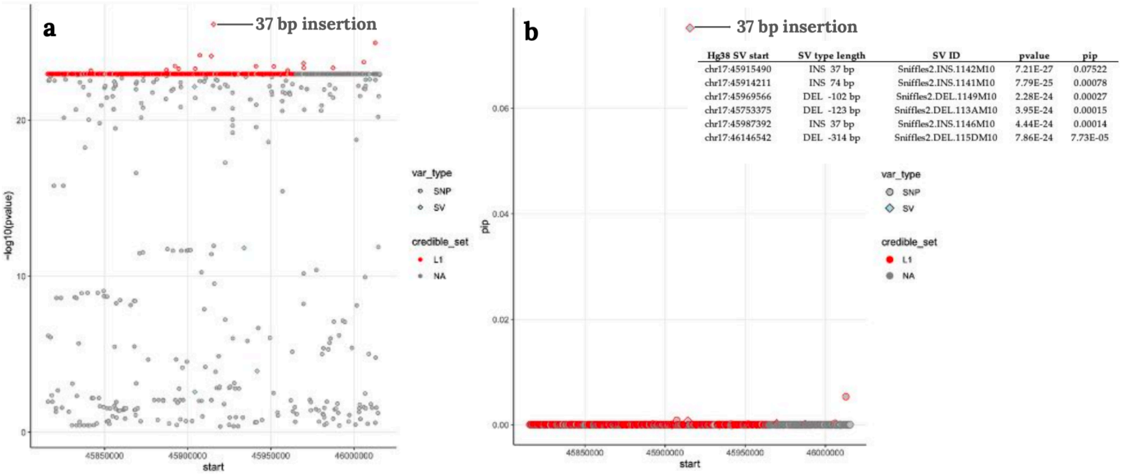


| Hg38 SV start | SV type length | SV ID | pvalue | pip |
|---|---|---|---|---|
| chr17:45915490 | INS 37 bp | Sniffles2.INS.1142M10 | 7.21E-27 | 0.07522 |
| chr17:45914211 | INS 74 bp | Sniffles2.INS.1141M10 | 7.79E-25 | 0.00078 |
| chr17:45969566 | DEL -102 bp | Sniffles2.DEL.1149M10 | 2.28E-24 | 0.00027 |
| chr17:45753375 | DEL -123 bp | Sniffles2.DEL.113AM10 | 3.95E-24 | 0.00015 |
| chr17:45987392 | INS 37 bp | Sniffles2.INS.1146M10 | 4.44E-24 | 0.00014 |
| chr17:46146542 | DEL -314 bp | Sniffles2.DEL.115DM10 | 7.86E-24 | 7.73E-05 |

**Supplementary Figure 9.** Fine-Mapping of eQTL for *LRRC37A2* in the ADRC Cohort. Example of fine-mapping of the eQTL for LRRC37A2 (ENSG00000238083) in the ADRC dataset, with the lead variant being a 37 bp insertion (Sniffles2.INS.1142M10, chr17:45915490). The L1 cluster includes variants within the credible set. Structural variants (SVs) are represented as diamonds, and single-nucleotide polymorphisms (SNPs) as circles. **a,** shows the –log10(p-value), and **b,**displays the posterior inclusion probability (PIP).
